# Enzymatically Polymerized Glycolated Conductive Polymers as Soft Electrodes for Neural Bioelectronic Interfaces

**DOI:** 10.1021/acsami.6c07522

**Published:** 2026-07-02

**Authors:** Luigi Fabiano, Tobias Abrahamsson, Ludovico Aloisio, Rémy Cornuéjols, Donghak Byun, Grazia Maria Lucia Messina, Xenofon Strakosas, Daniel T. Simon, Magnus Berggren, Chiara Musumeci

**Affiliations:** † Laboratory of Organic Electronics, Department of Science and Technology, 4566 Linköping University, 60174 Norrköping, Sweden; ‡ Department of Chemical Sciences, University of Catania and CSGI, Viale A. Doria 6, 95125 Catania, Italy; § Wallenberg Initiative Materials Science for Sustainability, Department of Science and Technology, Linköping University, 60174 Norrköping, Sweden

**Keywords:** ETE-based polymers, glycolated conducting polymers, enzymatic polymerization, organic mixed ionic−electronic conductors, electrochemical swelling, nanomechanics, neural biointerfaces, organic electrochemical transistors

## Abstract

Organic bioelectronics relies on materials capable of efficiently transducing signals between ionic biological environments and electronic devices. Conducting polymers are particularly attractive for this purpose due to their mixed ionic–electronic conductivity, mechanical compliance, and chemical tunability. Among them, bis-ethylenedioxythiophene-thiophene (ETE)-based polymers can be synthesized in situ via mild enzymatic reactions, enabling seamless and substrate-free integration with biological systems. Here, we investigate the impact of hydrophilic side-chain engineering on the physicochemical, electrochemical, and biological properties of ETE-based polymers by comparing two polymers which differ only by the presence of a triethylene glycol side chain between the ETE core and the terminal carboxylic group. We show that glycolation leads to increased film hydration and surface roughness without a measurable change in elastic modulus, suggesting competing effects from molecular ordering and ionic cross-linking. In a neuronal cell model, the glycolated polymer exhibits markedly enhanced cytocompatibility and cell adhesion, likely driven by its increased surface roughness and matrix topography. By combining electrochemical quartz crystal microbalance with dissipation monitoring, in-operando UV–vis spectroscopy, and electrochemical atomic force microscopy, we correlate ionic transport, swelling behavior, and nanomechanical responses, revealing enhanced electrochemically induced swelling in the glycolated polymer. Finally, when implemented as active channel materials in organic electrochemical transistors, both polymers display comparable performance, although the glycolated polymer shows slightly reduced cycling stability. These findings highlight the complex trade-offs introduced by side-chain glycolation and provide design guidelines for enzymatically synthesized conducting polymers in bioelectronic interfaces.

## Graphical abstract

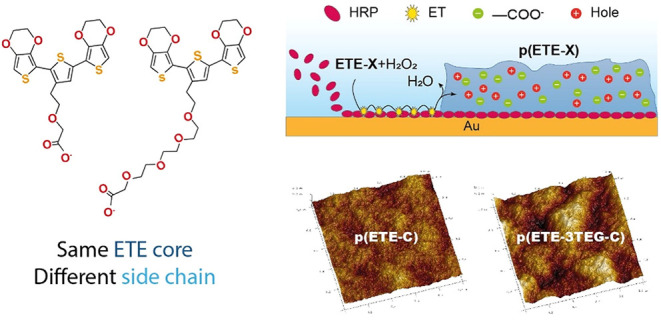

## Introduction

Organic bioelectronics aims to bridge the signaling electrolyte components of biology with the electronic domain of devices by exploiting materials capable of efficiently transducing signals between the two. Among these, conducting polymers have emerged as a leading candidate for neural interfaces, biosensors, and bioactuators owing to their mixed ionic–electronic conductivity, mechanical softness, and chemical tunability.^1,2^ Their ability to transport both ionic and electronic charge carriers supports operation in physiological environments, and can promote communication with cells and tissues, while maintaining low impedance and high charge storage capacity.

Bis-ethylenedioxythiophene-thiophene (ETE, EDOT-thiophene-EDOT)-based polymer electrolytes represent a particularly compelling class of conductive polymers because they can be synthesized in situ within tissues and cells through mild enzymatic reactions, due to their low oxidation potentials and water solubility, resulting in the formation of seamless and substrate-free bioelectronic interfaces.^3,4^ Their chemical structure allows straightforward incorporation of diverse side chains to tailor key properties, including film formation ability,^5^ aggregation behavior^6^ and device performance.^7^ Optimizing side chain chemistry is essential not only for efficient electronic and ionic transport, but also for controlling nanoscale structure, morphology, mechanical softness, and swelling behavior, properties that strongly influence interactions with biological systems. In addition, the chemical structure impacts adhesion and incorporation along and within biological surfaces and bulk, respectively. High water uptake facilitates ionic transport but can compromise mechanical robustness, while dense, less hydrated films hinder volumetric doping and reduce biocompatibility.^8,9^

The incorporation of hydrophilic side chains, such as oligoether side chains that can coordinate ions and water has proven to be an effective strategy to modulate ionic dynamics and mechanical softness simultaneously.^10,11^ Due to their low stiffness, glycolated polymers can in principle minimize adverse immune reactions^12^ and thus promote biocompatibility. However, during electrochemical cycling, ion and water insertion and removal can trigger major volume variations, which can have nontrivial effects when dealing with biointegration. On one side, these variations can substantially change the material’s stiffness, complicating its ability to remain mechanically compatible with the tissue under varying redox conditions. On the other hand, by effectively behaving as actuators they could bring new functionality to the bioelectronic interfaces, such as the ability to deliver different mechanical stimuli on demand, or to enable controlled drug delivery through electrochemically driven volume changes.

In this work, we compare two structurally related conjugated polymers, p­(ETE-C) and p­(ETE-3TEG-C), synthesized via surface-initiated enzymatic polymerization and differing only in the presence of a triethylene glycol (3TEG) side chain spacer between the ETE core and the terminal carboxylic group (Figure 1). We demonstrate that glycolation yields more hydrated and rougher films; however, this does not translate into a measurable difference in elastic modulus, suggesting the involvement of competing effects such as molecular ordering and ionic cross-linking. Using a neuronal cell model, we assess metabolic activity and cell adhesion and observe a markedly enhanced cytocompatibility of p­(ETE-3TEG-C), potentially arising from an increased surface roughness which, together with mechanical compliance, is known to promote neuronal adhesion and proliferation. Through a combined analysis using electrochemical quartz crystal microbalance with dissipation (EQCM-D), in operando UV–vis spectroscopy, and electrochemical atomic force microscopy (EC-AFM), we correlate ionic transport, swelling behavior, and nanomechanical responses, revealing enhanced electrochemically induced swelling and reduced stability in the glycolated polymer. Finally, we evaluated the electrical performance of the two materials in organic electrochemical transistors (OECTs), exhibiting comparable performance, with p­(ETE-3TEG-C) showing slightly reduced cycling stability under pulsed conditions.

**Figure 1:**
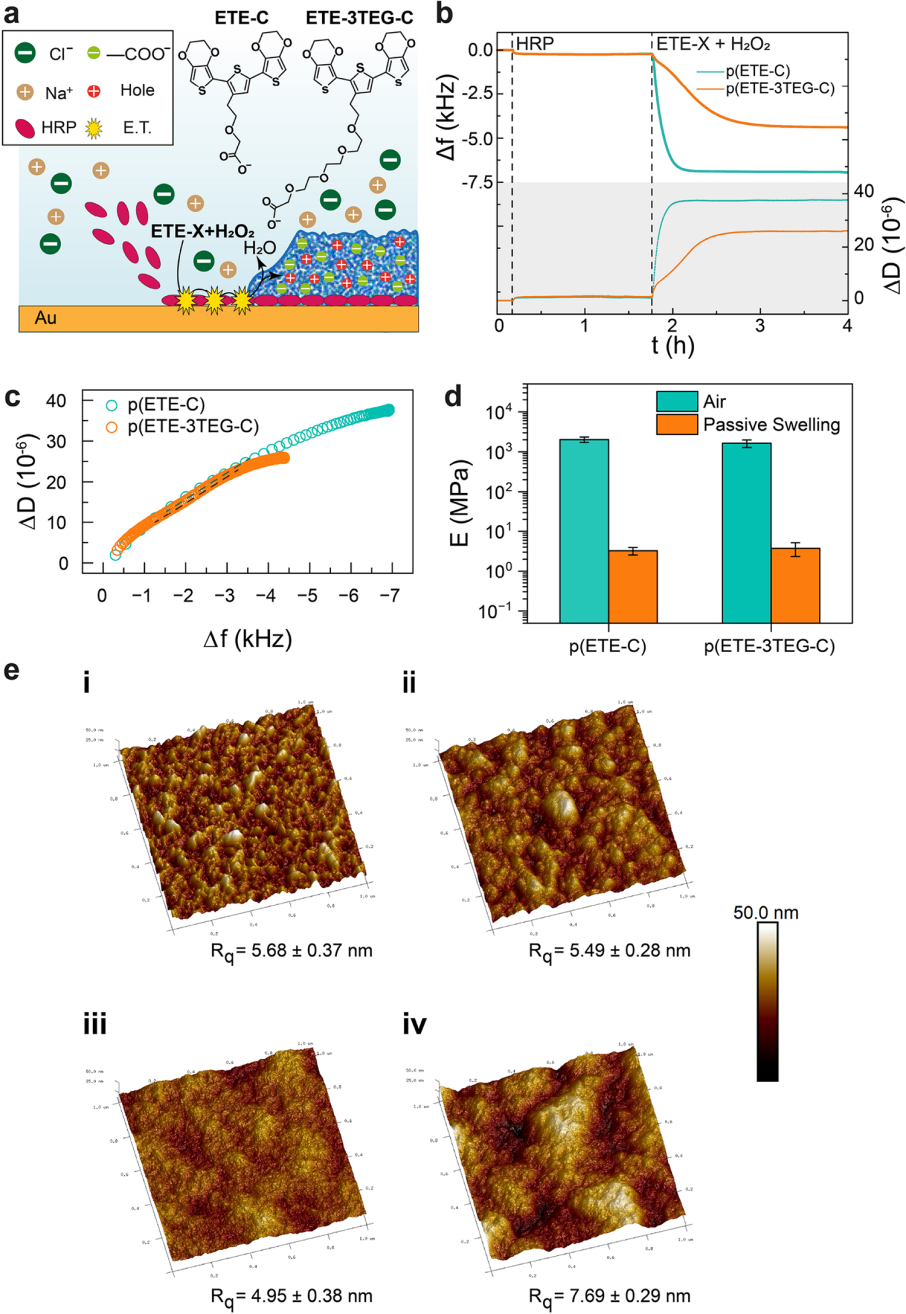
(a) Schematic illustration of the enzymatic polymerization mechanism of ETE-C and ETE-3TEG-C on Au, mediated by HRP in the presence of H_2_O_2_, including ion transport and charge compensation during film growth. Chemical structures of the monomers are shown on top. (b) QCM-D monitoring during the enzymatic polymerization of p­(ETE-C) and p­(ETE-3TEG-C), shown in teal and orange, respectively, performed in PBS at 37 °C. The shaded region highlights the portion corresponding to the D curves. (c) Df plot, focusing the polymerization region. Dashed lines indicate the region fitted to extract the slopes. (d) Young’s modulus of p­(ETE-C) and p­(ETE-3TEG-C) obtained in air (teal) and NaCl 0.1 M (orange); (e) AFM topography images of p­(ETE-C) (i-ii) and p­(ETE-3TEG-C) (iii, iv), acquired in air (i, iii) and in PBS (ii, iv). *R*_q_ values are reported in the images.

## Results and Discussion

### Polymers Synthesis

ETE-C and ETE-3TEG-C monomers were synthesized following the procedure reported in the Materials and Methods section. In situ enzymatic polymerization was performed using a horseradish peroxidase (HRP)/H_2_O_2_ system in aqueous buffer. HRP activates H_2_O_2_ to generate oxidizing equivalents that convert the monomers into radical intermediates, which subsequently couple to form the conjugated polymer network. This approach enables polymer formation under mild, physiologically compatible conditions without external electrochemical bias.

Figure 1 includes results from synthesizing p­(ETE-C) and p­(ETE-3TEG-C) along the surface of gold QCM-D sensors. In the first step, HRP is introduced in the cell and left adsorbing for 1 h; the concentration was chosen to ensure near saturation of the surface. Upon subsequent introduction of the monomer/H_2_O_2_ mixture, both systems formed a polymer film with rates of 795.79 ± 2.95 and 161.78 ± 0.07 ng cm^–2^ min^–1^ for p­(ETE-C) and p­(ETE-3TEG-C), respectively (Figure 1b, S7a and Table S1). The simultaneous decrease in frequency (Δ*f*) and increase in dissipation (Δ*D*) indicate continuous film growth.

The smaller absolute Δ*f* and lower Δ*D* values observed for p­(ETE-3TEG-C) indicate a reduced total mass uptake rather than a different viscoelastic response. In fact, the Δ*D*-Δ*f* region associated with the actual polymerization process (from monomer addition to film stabilization, Figure 1c) exhibits identical slopes for both materials (0.06 × 10^–6^ Hz^–1^, *R* ^2^ = 0.999). This demonstrates that the viscoelastic dissipation per unit of coupled mass is essentially the same during film growth. Therefore, the lower Δ*f* and Δ*D* amplitudes observed for p­(ETE-3TEG-C) mainly reflect a lower extent of polymerization rather than differences in mechanical softness of the resulting film (see also Figure S8 and Table S2). This interpretation is supported by the quantitative parameters summarized in Table 1, which show a lower polymer mass and polymerization yield for p­(ETE-3TEG-C), while the ΔD/Δf ratio remains identical for the two polymers.

**Table 1:** QCM-D Results of HRP-Assisted Polymerization on Gold-Coated Quartz Sensors^a^

polymer	HRP mass (ng cm^–2^)	polymer mass (μg cm^–2^)	polymerization yield (ng ng^–1^)	Δ*D*/Δ*f* (10^–6^ Hz^–^^1^)	*E* (GPa) in air	*E* (MPa) in NaCl 0.1 M
p(ETE-C)	655.06 ± 3.79	12.97 ± 0.04	19.75 ± 0.11	0.06	2.03 ± 0.32	3.24 ± 0.35
p(ETE-3TEG-C)	584.45 ± 17.10	8.34 ± 0.02	14.10 ± 0.41	0.06	1.64 ± 0.35	3.74 ± 1.40

a The table reports the mass of HRP adsorbate and the subsequently formed polymer layer, together with the calculated polymerization yield (polymer-to-HRP mass ratio) and the Δ*D*/Δ*f* ratio, which reflects the viscoelastic character of the resulting films (see also Figure S8 and Table S2), as well as Young’s moduli obtained in air and NaCl 0.1 M.

Because the polymers are generated directly as surface-bound films through enzymatic polymerization, conventional solution-based molecular-weight determination is not readily applicable; therefore, a contribution from differences in chain length or molecular weight cannot be completely excluded.

The Δ*D*-Δ*f* plots clearly reveal distinct kinetic regimes. More data points in the same interval means a slower process, while a lower amount of data points reflect a faster process. p­(ETE-C) progresses rapidly along the Δ*D*-Δ*f* path, maintaining an almost linear response that is consistent with a single dominant growth regime once monomer diffusion across the surface is established. In contrast, p­(ETE-3TEG-C) evolves more slowly and shows a much higher density of data points, ultimately reaching stabilization earlier. This behavior may reflect an interplay between aggregation and diffusion-related effects in the triethylene glycol-substituted system. In particular, we argue that the change in slope observed in Figure 1b (Δ*f*) after approximately 2 h may indicate that, upon reaching a critical aggregation threshold, ETE-3TEG-C undergoes accelerated film formation. The hydrophilic and sterically bulky TEG side chains promote increased hydration but hinder efficient monomer packing and propagation, leading to the formation of a thinner and lighter polymer film.

AFM topography (Figure 1e) further corroborates these findings. Bare gold and HRP-coated substrates show the expected fine-grained texture (Figure S7b,c), whereas polymer deposition results in distinct morphologies. p­(ETE-C) (Figure 1e,i-ii) forms a compact, finely textured surface composed of small polymer grains and displays stable roughness upon hydration (*R*_q_ = 5.68 ± 0.37 nm in air, 5.49 ± 0.28 nm in PBS, *p* = 0.39). In contrast, p­(ETE-3TEG-C) (Figure 1e,iii-iv) exhibits a more clustered morphology with larger domains in air and a marked increase in roughness after immersion in PBS (*R*_q_ = 4.95 ± 0.38 nm in air, 7.69 ± 0.29 nm in PBS, *p* < 0.001), indicating swelling and reorganization of the hydrated polymer matrix.

Despite their different hydration behavior, the Young’s moduli of the two polymers remain comparable (in the order of few MPa) (Figure 1d). The fact that p­(ETE-3TEG-C) undergoes expansion while retaining similar stiffness suggests a partial decoupling between swelling and mechanical softening. This observation is consistent with the framework proposed in recent literature,^13,14^ where competing processes—ion/solvent plasticization versus oxidation-driven backbone ordering and ionic cross-linking through polaron–counterion interactions, jointly govern the overall mechanical response. Young’s moduli of the two polymers in air and 0.1 M NaCl are reported in Table 1.

### Cell-Polymer Interfaces

To evaluate the cytocompatibility of the p­(ETE-3TEG-C) and p­(ETE-C) polymeric substrates and determine their ability to support neuronal adhesion and growth, we used F11 cells, a hybrid cell line derived from the fusion of embryonic rat DRG cells and a mouse neuroblastoma cell line (N18TG2), commonly used to study various aspects of neuronal function and physiology. We assessed both metabolic activity and cell attachment over time. The AlamarBlue viability assay showed comparable metabolic activity across all substrates at 24 h, indicating similar initial adhesion after seeding (Figure S9). To better evaluate differences in cell behavior over time, fluorescence values at 48 h were normalized to the corresponding 24 h measurements (Figure 2a). This representation highlighted that both polymeric substrates supported F11 viability and proliferation, with p­(ETE-3TEG-C) yielding the highest metabolic activity among the tested materials outperforming PDL, a widely used standard coating used to promote F11 cells adhesion. Live/Dead staining with Calcein AM and EthD 1 (Figure 2b) confirmed low cytotoxicity across all conditions, with high viability percentages in all groups (Glass: 96.4%, PDL: 93.5%, p­(ETE-C): 91.4%, p­(ETE-3TEG-C): 98.0%, more details in Table S3). However, the modest differences observed between conditions should not be used for quantitative comparisons between groups in adherent cultures, as dead or compromised cells can detach during handling and may be underrepresented in end point images. Nevertheless, confocal microscopy revealed a marked difference in the density of attached cells, with p­(ETE-3TEG-C) again showing visibly higher cell coverage, in agreement with the AlamarBlue results. The enhanced biocompatibility of this polymer may arise from its mechanical compliance and increased surface roughness, both of which are known to promote neuronal adhesion and proliferation.

**Figure 2:**
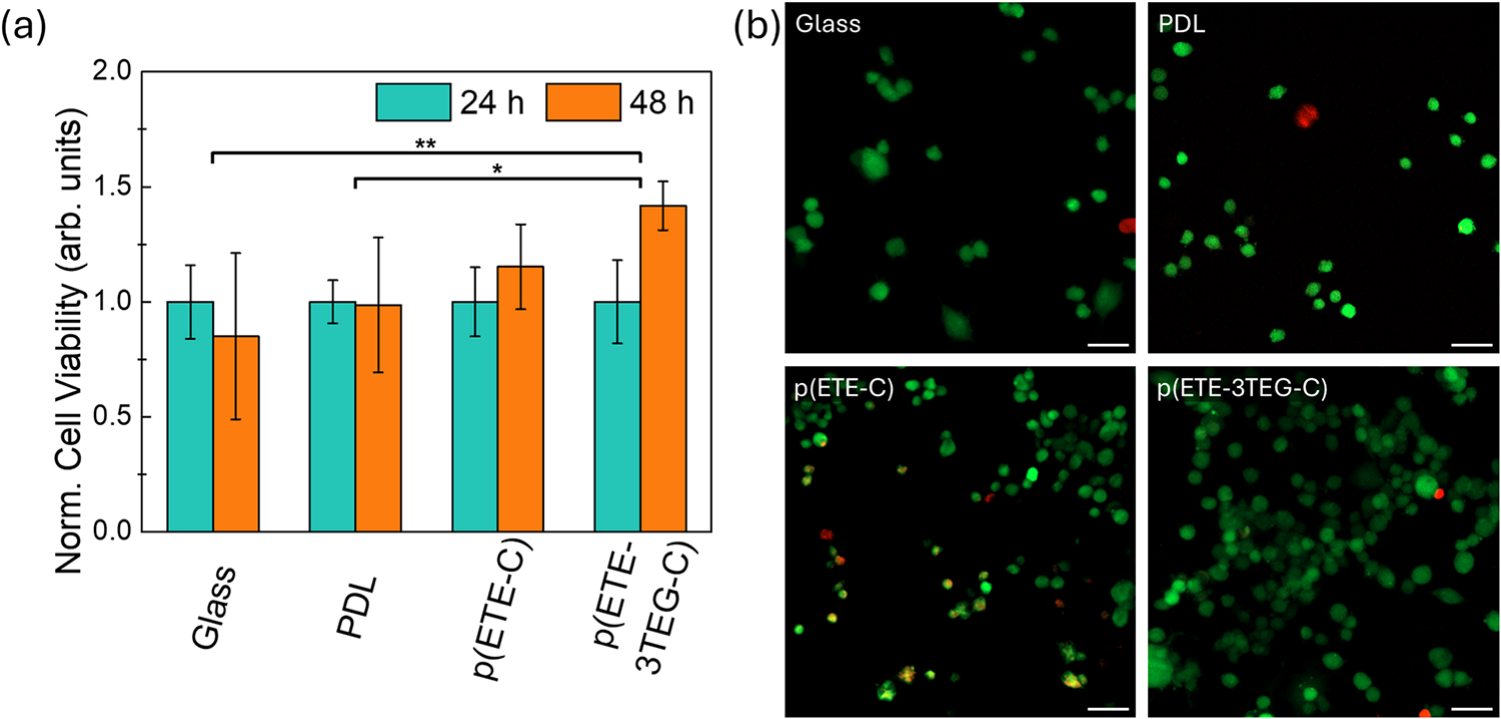
(a) Normalized emission intensity at 600 nm (excitation wavelength: 546 nm) of AlamarBlue after 3 h incubation with F11 cells in complete culture medium, measured at 24 and 48 h after seeding on Glass, PDL, p­(ETE-C), and p­(ETE-3TEG-C). Signal was normalized to the corresponding 24 h value for each substrate (24 h = 1, arbitrary units). Data are reported as mean ± SD (*N* = 7 per condition from 3 independent substrate preparations). *p < 0.05; **p < 0.01; one way ANOVA followed by Bonferroni’s or Scheffe’s post hoc tests. (b) Representative confocal live/dead images of F11 cells on Glass, PDL, p­(ETE-C), and p­(ETE-3TEG-C), stained with calcein AM (green, viable cells) and ethidium homodimer 1 (red, nonviable cells). Scale bars: 50 μm.

### Mass Uptake and Ion Exchange

To examine how the properties of the two polymers evolve under redox cycling, we performed correlated EQCM-D, *in-operando* UV–vis-NIR spectroscopy and EC-AFM (Figure 3). Under cyclic bias (Figure 3a), both films display predominantly capacitive behavior in PBS, yet the QCM-D traces reveal a clear ion-coupled swelling response: upon oxidation the films contract (mass decreases), while upon reduction they swell (mass increases), with reproducible amplitudes over consecutive cycles. The optical spectra in Figure 3b follow the expected evolution, with the neutral π–π* transition decreasing and the polaronic absorption increasing during oxidation and reversing during reduction. The corresponding doping index (Figure 3d) rises and falls accordingly, with p­(ETE-3TEG-C) consistently reaching a higher doping level at matched potential.

**Figure 3:**
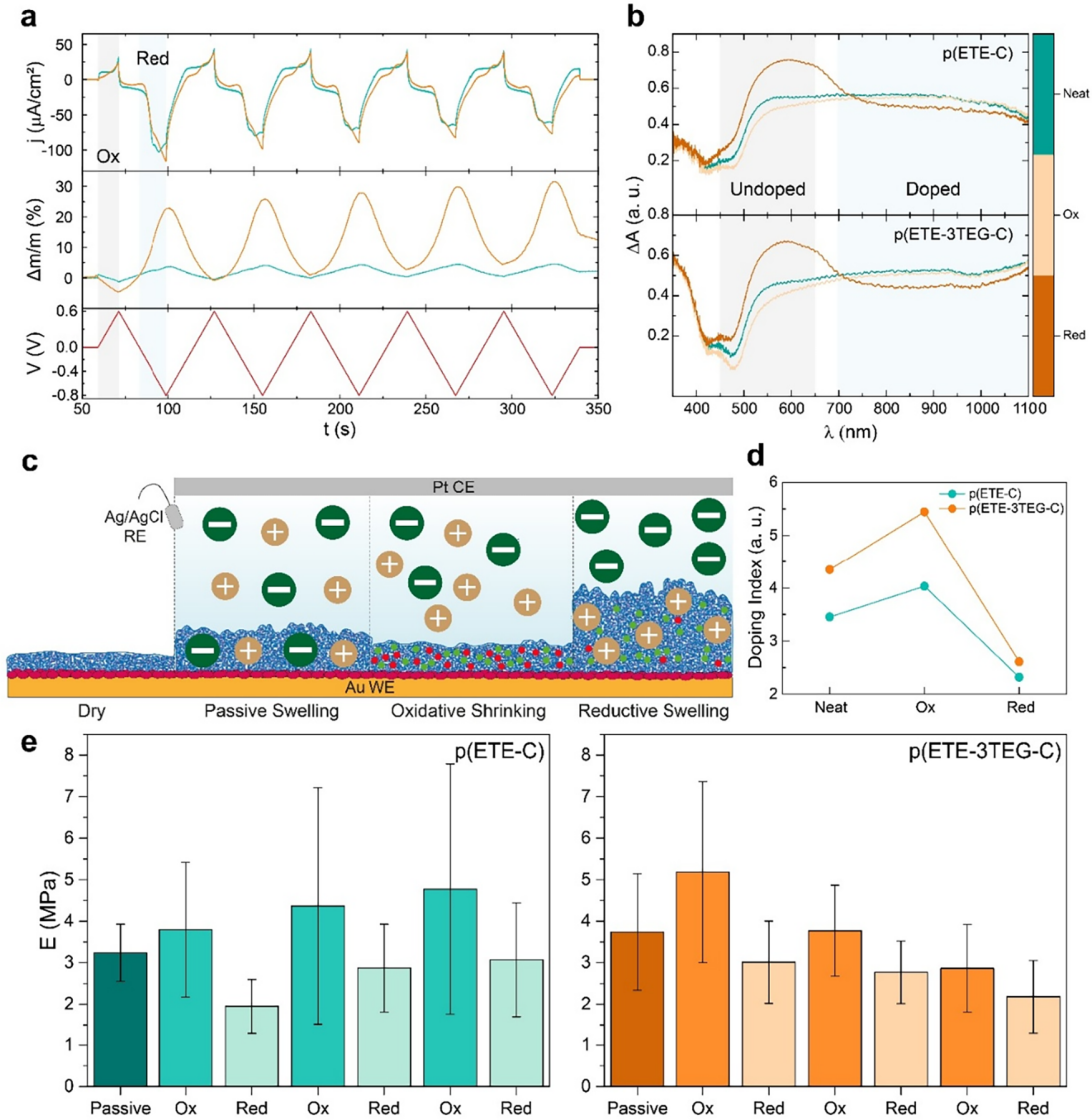
*In-operando* spectroelectrochemistry and EQCM-D swelling of p­(ETE-C) (teal) and p­(ETE-3TEG-C) (orange) in NaCl 0.1 M at 37 °C. (a) Cyclic Voltammetry (bottom, −0.8 to +0.6 V vs Ag/AgCl, 50 mV/s) with the corresponding current density *j* (top) and relative mass change from QCM-D, Δ*m*/*m* (middle) calculated referring to the reached mass after stabilization (passive swelling) (*m*_Neat_), by using the VE model. Shaded bands highlight Ox (gray) and Red (light blue) of a representative CV cycle. (b) *In-operando* UV–vis-NIR differential spectra Δ*A*(λ) acquired for three electrochemical states (Neat, Ox at +0.5 V, Red at −0.5 V) for each polymer. Shaded vertical windows mark the neutral π–π* region (gray, 450–650 nm) and the polaronic band (light blue, 700–1100 nm). (c) Schematic of the experimental configuration (Au coated-working electrode, Pt counter, Ag/AgCl reference) and qualitative picture of the passive swelling, oxidative shrinking, and reductive swelling processes, with exchange of ions and water between film and electrolyte. (d) Doping index extracted from the spectra as discussed and plotted for the three different states. p­(ETE-3TEG-C) yields a systematically higher DI than p­(ETE-C), consistent with a larger relative polaronic contribution under identical conditions; (e) Young’s modulus (mean value ± SD) of p­(ETE-C) and p­(ETE-3TEG-C) extracted from the force–distance curves (*n* = 300) collected in NaCl 0.1 M for each electrochemical step. Representative force curves for each electrochemical state and the derived Young’s moduli are reported in Figure S10 and Table S4.

Despite forming a lighter film, p­(ETE-3TEG-C) displays a substantially larger swelling response, indicating that ion accessibility through the side-chains governs its electrochemically active volume. These changes are related to the movement of water-coupled ions in and out of the polymer films (Figure 3c). Once the films are immersed in electrolyte, the carboxylate end groups interact with the positive charges already present due to self-doping during polymerization. Consequently, no significant ion uptake occurs initially, and the observed passive swelling is mainly due to ion pairs and bound water.^15^ Under positive bias, cations are expelled, leading to contraction; under negative bias, cation uptake (with associated water) increases while previously absorbed chloride ions are likely repelled. This redistribution reflects the higher doping levels achieved and the need to maintain electroneutrality, which stabilizes the polaronic absorption band.^16–18^

The normalized mass modulation is markedly larger for p­(ETE-3TEG-C) than for p­(ETE-C), revealing stronger water/ion exchange per cycle (Table 2). This dynamic behavior correlates with the growth data, where p­(ETE-3TEG-C) deposited less mass but retained similar viscoelastic properties. Hence, the triethylene glycol side chains enable more extensive charge and solvent movement for a given potential excursion.

**Table 2:** Mass Uptake Percentage during the CV Scan (Red Refers to Values Obtained Once −0.5 V is Reached, while Ox when +0.5 V is Applied to the Film), Reported as Mean Value ± SD

polymer	Δ*m*/*m* Red (%)	Δ*m*/*m* Ox (%)
p(ETE-C)	3.47 ± 0.48	0.56 ± 0.26
p(ETE-3TEG-C)	22.10 ± 4.00	3.21 ± 1.55

The gradual increase in the Δ*m*/*m* baseline observed for p­(ETE-3TEG-C) during repeated redox cycling is likely associated with the incomplete reversibility of water and ion exchange within the glycolated polymer network. Despite this baseline drift, the persistence of a clear redox-coupled mass response over successive cycles indicates that film remains electrochemically active. The 3TEG side chains promotes the uptake of water and solvated ions, some of which may be retained within the swollen polymer matrix on the time scale of the electrochemical cycle. The progressive accumulation of this residual hydrated fraction may therefore account for the observed increase in the apparent hydrated mass baseline.

Despite the different swelling amplitudes, the Young’s moduli of the two polymers remain comparable (few MPa) and follow the same trend: stiffening upon oxidation and softening upon reduction (Figure 3e). The apparent progressive decrease in the Young’s modulus of p­(ETE-3TEG-C) during cycling is consistent with this interpretation and suggests gradual plasticization and structural relaxation of the hydrated polymer network under repeated ion/water uptake.

Thus, glycolation primarily enhances the swelling capacity and electrochemically accessible volume without compromising mechanical robustness. The resulting films combine large reversible volume changes with a narrow modulus range, and desirable property for stable, hydrated conjugated polymer electrodes.

Although p­(ETE-3TEG-C) deposits less mass than p­(ETE-C) at the end of enzymatic polymerization (0.88 × 10^4^ vs 1.36 × 10^4^ ng cm^–2^) and the two films exhibit nearly identical *f*/*D* ratios during growth (comparable viscoelastic quality), the glycolated film consistently displays larger reversible electroswelling over the same potential range.

To evaluate the electrical properties of the two polymer films, we fabricated organic electrochemical transistors (OECTs) using p­(ETE-C) or p­(ETE-3TEG-C) as the active layer (Figure 4). Both polymers exhibited accumulation-mode behavior, with channels doping under negative gate bias (Figure 4c,d). The maximum transconductance occurred at closely spaced gate voltages (≈0.3–0.5 V), reflecting similar operating points. Electrochemical impedance spectroscopy (EIS) measurements taken through the channel showed comparable impedance spectra for both polymers, indicating similar ionic/electronic coupling within the devices (Figure 4a,b).

**Figure 4:**
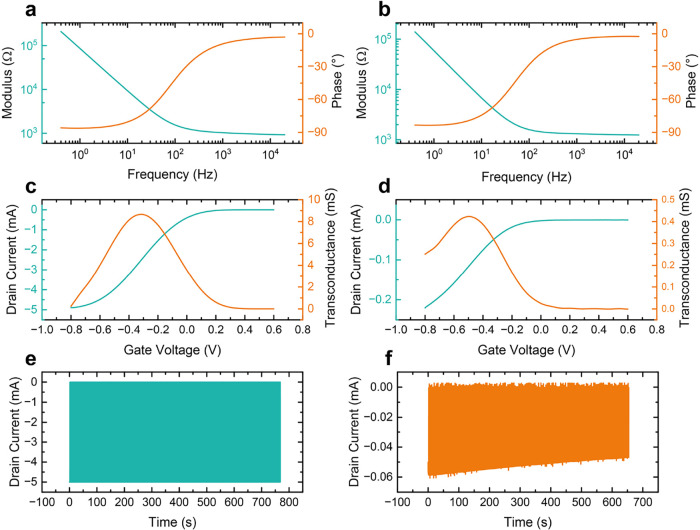
Electrical characterization of OECT devices based on p­(ETE-C) (left column) and p­(ETE-3TEG-C) (right column), fabricated with PLL as an adhesive layer. (a, b) Electrochemical impedance spectroscopy (EIS): modulus (left axis, teal) and phase (right axis, orange) as a function of frequency. (c, d) Transfer characteristics (drain current vs gate voltage, left axis, teal) and corresponding transconductance (right axis, orange). (e, f) Operational stability under pulsed gate bias (−0.5/+0.5 V): time-dependent drain current. The corresponding devices fabricated without PLL as an adhesive layer are reported in Figure S11.

However, devices based on both materials showed poor operational stability upon repeated cycling (Figure S11e,f). Modifying the fabrication protocol by introducing a poly-l-lysine (PLL) interlayer as an adhesion promoter significantly improved the cycling stability of p­(ETE-C)-based devices (Figure 4e). In contrast, OECTs fabricated with p­(ETE-3TEG-C) displayed reduced stability (Figure 4f), which may be attributed to the increased swelling of this polymer under operating conditions.

## Conclusion

In this work, we investigated the role of side-chain glycolation in enzymatically synthesized ETE-based conducting polymers by comparing p­(ETE-C) and p­(ETE-3TEG-C), two materials differing only by the presence of a triethylene glycol side chain. By combining surface-initiated polymerization, in-operando electrochemical and spectroscopic techniques, nanoscale mechanical characterization, and biological assessment, we establish a comprehensive structure–property-function relationship relevant to organic bioelectronic interfaces. We show that glycolation enhances ionic accessibility and expands the electrochemically addressable volume in ETE-based OMIECs. Compared to p­(ETE-C), the glycolated p­(ETE-3TEG-C) deposits less mass yet undergoes larger and reversible electroswelling and reaches a higher doping level at matched potentials as quantified from the optical spectra.

From a biological perspective, the glycolated polymer demonstrates superior cytocompatibility and supports enhanced neuronal cell adhesion and proliferation, consistent with its increased surface roughness.

When implemented as active channel materials in organic electrochemical transistors, both polymers show comparable electrical performance, with the glycolated system displaying slightly reduced cycling stability, reflecting the trade-off between enhanced ionic accessibility and long-term structural robustness. Collectively, these findings establish design principles for enzymatically synthesized conducting polymers used in bioelectronic interfaces.

## Materials and Methods

### Materials

ETE-C and ETE-3TEG-C monomers were synthesized from 2-(2,5-dibromothiophen-3-yl)­ethan-1-ol through a modular functionalization–coupling strategy. In the first stage, the pendant hydroxyl group was derivatized to introduce the desired side-chain architecture. Direct O-alkylation with methyl bromoacetate via a Williamson ether synthesis afforded the corresponding ether bearing a terminal methyl ester. Alternatively, a triethylene glycol (3TEG) spacer was installed through nucleophilic substitution with 2-(2-(2-(trityloxy)­ethoxy)­ethoxy)­ethylmethanesulfonate, followed by trityl deprotection to liberate the terminal hydroxyl group and subsequent O-alkylation with methyl bromoacetate to introduce the methyl ester functionality. In the second stage, the conjugated backbone was constructed via a double Suzuki cross-coupling reaction at the 2,5-dibromo positions, attaching EDOT units to afford the EDOT-thiophene-EDOT framework. Finally, hydrolysis of the methyl ester groups yielded the corresponding carboxylate-functionalized ETE monomers (Figure 1a). Detailed synthetic procedures and characterization data are provided in the Supporting Information (Scheme 1 and Figures S1–S6).

Horseradish peroxidase (HRP, Type I, ≥50 units/mg) and phosphate buffered saline (PBS) tablets were purchased from Sigma-Aldrich. A 35 wt % hydrogen peroxide (H_2_O_2_) aqueous solution was obtained from Acros Organics. Stock solutions were prepared at concentrations of 1 mg mL^–1^ for the monomer and HRP, and 1 mM for H_2_O_2_, using PBS (10 mM phosphate buffer, 137 mM NaCl, 2.7 mM KCl, pH 7.4 at 25 °C) as solvent. Freshly diluted aliquots were prepared in PBS immediately prior to each experimental session.

Unless otherwise specified, all experiments were conducted at room temperature (22 °C).

### Polymer Films Deposition

P­(ETE-C) and P­(ETE-3TEG-C) polymer films were obtained by enzymatic polymerization of ETE-C and ETE-3TEG-C on gold substrates following a procedure adapted from previous works.^19,20^

For QCM-D experiments, gold crystal sensors were used as substrates. The polymerization was carried out by sequential injection of enzyme, monomer and H_2_O_2_ solutions into the flow cell of the EQCM-D apparatus, HRP (1 mg mL^–1^ in PBS, pH 7.4) was introduced in a volume of 450 μL at a flow rate of 150 μL min^–1^ and allowed to adsorb for 1 h at 37 °C. Following adsorption, the sensors were rinsed twice with PBS (15 min each). Subsequently, a 0.1 mg mL^–1^ monomer solution (ETE-C or ETE-3TEG-C in PBS), supplemented with 1 mM H_2_O_2_ was injected under the same conditions and left polymerizing for 2 h. Again, two consecutive PBS washes of 15 min each were performed before a final rinse with ultrapure water.

For EC-AFM and cell studies gold coated glass slides were used as substrates, and the film was prepared by sequential adsorption of enzyme, monomer and H_2_O_2_. HRP was first drop-cast from a 1 mg mL^–1^ PBS solution and incubated for 1 h at 37 °C, followed by two rinsing steps with PBS. The monomer (ETE-C or ETE-3TEG-C, 0.1 mg mL^–1^ in PBS, supplemented with 1 mM H_2_O_2_) was then drop-cast and allowed to adsorb and polymerize for 2 h at 37 °C, after which the substrates were rinsed twice with PBS and finally with ultrapure water.

### Devices Fabrication

OECTs were fabricated with the following protocol. Prior to starting fabrication, glass slides used as substrates were sequentially cleaned with Decon90 (Micro90), acetone, and isopropanol. Metal patterns, including electrodes, contact pads, and feed lines, were defined by a lift-off process, using S1818 photoresist, an MA-6 SUSS mask aligner, and MF-319 developer. After deposition of Cr and Au (5 and 40 nm, respectively) onto the substrate using a thermal evaporator, the lift-off process was carried out in an acetone bath. A 1 μm thick parylene C layer was then deposited with an adhesion promotor (A-174) to insulate the interconnections by chemical vapor deposition. A soap solution (Micro90) diluted to 2% was spin-coated as a release layer, followed by deposition of a 3 μm thick parylene C film. A thick photoresist film (AZ10XT (520 cP)) was photolithographically patterned to define the openings for contact pads and electrodes. The parylene films were then etched by reactive ion etching at 200 W with CF_4_/O_2_ gas flow rate of 20 and 100 sccm under a vacuum pressure of 20 Pa. Finally, the remaining photoresist used as an etch mask was removed with acetone and isopropanol.

Polymer deposition was performed according to the procedure used for EC-AFM and cell studies. A modified procedure was used to improve cycling stability which involved the deposition of a poly-l-lysine (PLL) as an adhesion layer (2 μL, 0.1 wt % (mw: 150–300 kDa)), followed by a drying step (30 min) in ambient and a rinsing step with DI water. HRP (2 μL, 0.2 mg mL^–1^) was then applied and let it dry in ambient for 30 min. Finally, the monomer/H_2_O_2_ solution (5 μL, 1 mg mL^–1^ monomer, 1 mM H_2_O_2_) was applied and left to polymerize for 1 h. The solution was then removed, and the film rinsed with DI water. The top parylene layer was finally peeled off before measurements.

### Quartz Crystal Microbalance with Dissipation Monitoring (QCM-D)

QCM-D experiments were performed using a QSense E4 Analyzer (Biolin Scientific) equipped with a high-precision multichannel IPC pump (ISMATEC). The resolution in *F* and *D* was 0.1 Hz and 1 × 10^–7^,^21^ respectively. Titanium/Gold-coated quartz sensors (5 MHz, Biolin Scientific) were mounted inside standard flow modules and cleaned for 30 min in an ultraviolet-ozone cleaner (Novascan Technologies) to remove surface contaminants and enhance hydrophilicity.

Frequency and dissipation shift Δ*f_n_* and Δ*D_n_* (overtones *n* = 3, 5, 7, 9, 11 which corresponding frequencies are 15, 25, 35, 45, 55 MHz) were analyzed in QSense Dfind 1.2.7.

Mass uptake was obtained from (i) Sauerbrey relation in regimes where the film behaved as rigid and uniform, i.e., for small dissipation changes (Δ*D* < 2 × 10^–6^) and linear Δ*D*-Δ*f* correlation, conditions under which viscoelastic and coupling effects can be neglected (e.g., HRP adsorption), or (ii) with the Viscoelastic (VE) Voigt model otherwise.

According to Sauerbrey (i),^22^ the adsorbed mass variation (Δ*m*) was calculated from the frequency shift (Δ*f*) using the eq 1

1 $$\Delta m=-C\frac{\Delta f_{n}}{n}$$

where C is the crystal sensitivity constant (17.7 ng cm^–2^ Hz^1–^ for a 5 MHz crystal) and *n* is the overtone number.

The viscoelastic (ii) Voigt model (VE, Voinova thin-film formulation)^23^ was used in case of non-negligible dissipation changes. Film thickness *h*_f_, areal mass Δ*m*/*A* = ρ_f_h, and viscoelastic parameters μ, η, which are the shear modulus and viscosity respectively, and can be extracted by the VE model, assuming a laterally homogeneous, no-slip film characterized by complex shear modulus described by eq 2

2 $$G^{*}\left(\omega \right)=G^{\prime }+iG^{\prime \prime }=\mu +i\omega \eta$$

Where G* is the complex shear modulus, *G′* is its storage component and *G*″ its loss component (storage and loss modulus), while ω is the angular frequency.

### Electrochemical Quartz Crystal Microbalance with Dissipation Monitoring (EQCM-D)

For combined electrochemical and QCM-D experiments, a μAutolab III potentiostat (Metrohm) was connected to the EQCM-D setup. The three-electrode configuration consisted of the Au–Ti-coated quartz crystal as working electrode, an external Ag/AgCl electrode (Dri-ref-2SH, World Precision Instruments) as reference electrode, and the Pt plate of the module as counter electrode. Prior to each measurement, a stable PBS baseline was established for at least 10 min.

QCM-D data collection was conducted by mean of QSoft, while modeling and fitting through QSense Dfind 1.2.7.

Cyclic voltammetry (CV) was performed in PBS and 0.1 M NaCl, scanning from −0.8 to +0.6 V vs Ag/AgCl at 50 mV s^–1^, starting from 0 V vs *V*_ref_. Multistep chronoamperometry (MSCA) was performed by alternating −0.5 and +0.5 V steps for 5 min each. Measurements were performed at 37 °C.

Capacitance was extracted from the last 3 full CV cycles after stabilization. With scan rate ν and a voltage span Δ*V*, the related capacitance can be calculated as reported in eq 3

3 $$C=\frac{\oint i\left(V\right)dV}{2\nu \Delta V}$$

The areal capacitance (*C*_A_) was evaluated by using geometric area *A* of the crystal sensor. CV data acquisition was made by Nova 2.1, data treatment by means of Origin 2024.

### In Operando UV–vis-NIR Spectroelectrochemistry

Optical spectra were recorded simultaneously with EQCM-D using an AvaSpec-2048CL-EVO-VA-50 spectrometer (Avantes) connected to the flow module via a bifurcated reflection probe (FCR-7UVIR400–2-BX, Avantes) and a halogen light source (AvaLight-HAL-S-Mini2, Avantes). Spectra were acquired in the 340–1100 nm range (integration time 500 μs, averaging of 100 acquisitions) and corrected against the reference spectrum of the bare crystal immersed in electrolyte. Data acquisition was performed with AvaSoft (Avantes). Differential spectra Δ*A*(λ) were computed with the same optical path and medium reference. To quantify the oxidation state in a thickness-independent manner, a *Doping Index* was defined as represented by eq 4

4 $$\mathrm{DI}=\frac{\int_{700}^{1100}\Delta A\left(\lambda \right)d\lambda }{\int_{450}^{600}\Delta A\left(\lambda \right)d\lambda }$$

where the band at 450–600 nm corresponds to the π–π* transition and the absorption at 700–1100 nm constitutes the polaronic band.^16,24^ Spectra were interpolated onto a common wavelength grid and integrated by the trapezoidal rule. When comparing data sets, identical reference, windows, and grid were enforced.

### Electrochemical Atomic Force Microscopy (EC-AFM)

EC-AFM experiments were performed on a Dimension Icon XR (Bruker).

Samples were mounted in a liquid electrochemical cell connected to a CHI760E bipotentiostat and filled with the electrolyte of interest. A Pt wire served as counter electrode and Ag/AgCl pellet as reference electrode.

For mechanical characterization, the deflection sensitivity was calibrated on sapphire substrates in electrolyte, and the cantilever spring constants were determined using the thermal noise method. Force–displacement curves were acquired under controlled loads (≥300 curves per sample, maximum load 10 nN) by recording approach-retraction cycles. The Young’s modulus was extracted by fitting the indentation data to the Derjaguin–Muller–Toporov (DMT) model, as reported in eq 5 ^25,26^

5 $$F+F_{\mathrm{ad}}=\frac{4\cdot E}{3\left(1-\nu^{2}\right)}\sqrt{R}\cdot \delta^{3/2}$$

where *F* is the applied force, *F*_ad_ the adhesion force, *E* the sample’s Young’s modulus, ν the Poisson’s ratio of the sample (0.3),^27,28^ *R* the probe radius, and δ the indentation depth.

AFM imaging was performed in PeakForce-QNM both in air and in PBS electrolyte, with images collected at different scales (1 × 1 μm^2^, 512 × 512 pixels with a scan speed of 1 Hz), to characterize morphological properties of such polymers, together with bare gold and HRP adsorbate on gold for comparison.

### Cell Culture Maintenance

F11 cells (cell line derived from rat DRG neurons and mouse neuroblastoma cells) were a gift from C. Svensson (Karolinska University, Stockholm, Sweden) and were maintained according to standard procedures. Cells were cultured in T-75 flasks using Roswell Park Memorial Institute (RPMI) 1640 medium supplemented with 15% fetal bovine serum, HAT supplement, and 200 μg mL^–1^ l-proline. Cultures were kept in a humidified incubator at 37 °C with 5% CO_2_. The medium was refreshed every 2–3 days, and cells were passaged weekly to keep confluency below 75%. For routine splitting, the culture medium was removed, and cells were incubated with PBS/EDTA (0.7 mM) for 2 min at 37 °C to facilitate detachment. Fresh medium was then added back to dilute the EDTA and stop dissociation, and the resulting cell suspension was centrifuged at 200 rcf for 5 min at room temperature. After centrifugation, cells were resuspended in fresh medium and reseeded into new T-75 flasks at a density of 5 × 10^4^ cells/mL in 10 mL of culture medium. For experiments, cells were seeded at 4000 cells cm^–2^ onto sterilized substrates and allowed to adhere and grow for at least 24 h before subsequent analysis.

### Viability Assay

To evaluate the biocompatibility of the polymeric substrates, a cell viability assay based on the AlamarBlue metabolic indicator was performed. This method quantifies cellular activity by measuring the reduction of resazurin, a nonfluorescent and cell-permeable compound, into resorufin, which exhibits strong fluorescence. The extent of this redox conversion, associated with a spectral shift in absorbance from 600 to 570 nm, reflects the number of viable and metabolically active cells on each substrate. F11 cells were seeded at a density of 4000 cells cm^–2^ onto four different substrates: glass, PDL-coated glass, p­(ETE-C), and p­(ETE-TEG-C). The PDL coating was applied 24 h before seeding to the relevant control samples. Cell viability and proliferation were assessed after 24 and 48 h from seeding. For each time point, the AlamarBlue reagent (Invitrogen, DAL1100) was diluted 1:10 in phenol-red-free medium and incubated with the samples for the duration specified in the manufacturer’s protocol. Medium without cells served as blank control. After incubation, aliquots of the supernatant were transferred to a 96-well plate, and fluorescence was recorded using an excitation wavelength of 560 nm and an emission wavelength of 600 nm. Each measurement was repeated three times to ensure reproducibility. To better highlight proliferation dynamics, fluorescence values obtained at 48 h were normalized to the corresponding 24 h measurements for each substrate, allowing changes in cell viability over time to be more clearly visualized.

### Confocal Imaging

Confocal imaging was performed to qualitatively assess cell viability and morphology on the different substrates. A standard Live/Dead assay was carried out using Calcein-AM (Calcein acetoxymethyl ester) to label viable cells and EthD-1 (ethidium homodimer-1) to mark membrane-compromised cells. After removing the culture medium, samples were gently rinsed with warm PBS. A staining solution containing 2 μM Calcein-AM and 4 μM EthD-1 diluted in PBS was freshly prepared and added to each sample, followed by a 20 min incubation at 37 °C in the dark. After staining, samples were washed once with PBS to remove excess dye before imaging. Confocal images were acquired using a Zeiss LSM980 inverted microscope equipped with 5× and 20× objectives. Calcein-AM was excited at 488 nm, and fluorescence was collected between 490–550 nm, while EthD-1 was excited at 561 nm and its emission detected between 650–700 nm to avoid spectral overlap. This configuration enabled both large-area overviews and higher-resolution assessment of cell attachment and viability across the different substrates. The acquired images were analyzed using Fiji (ImageJ).

### Statistical Analysis

Statistical comparisons were performed using Python. Normality and homoscedasticity were evaluated prior to each test. Depending on variance equality, either Student’s or Welch’s *t* tests were applied for pairwise comparisons, while one-way ANOVA was used to assess reproducibility across cycles. Significance thresholds were set as *p* < 0.05 (*), < 0.01 (**), < 0.001 (***), and <0.0001 (****). All AFM mechanical values were obtained from ≥300 independent force curves per electrochemical state.

## Supplementary Material



## Notes

Monomer synthesis and NMR characterization; enzymatic polymerization kinetics; additional QCM-D and viscoelastic modeling data; AFM topography of Au, HRP-coated substrates, and polymer films; cell viability and Live/Dead quantification; EC-AFM force spectroscopy and redox-dependent Young’s modulus analysis; and OECT characterization without PLL adhesive layer (PDF)
