# Supplementary material for: Enzymatically Polymerized Glycolated Conductive Polymers as Soft Electrodes for Neural Bioelectronic Interfaces
Source: ACS Appl Mater Interfaces. 2026 Jul 2;18(28):38502–11. doi: 10.1021/acsami.6c07522 (PMC13397480; doi:10.1021/acsami.6c07522)
Supplement: Supplementary file 1 [file am6c07522_si_001.pdf]

Supporting Information

Enzymatically polymerized glycolated conductive polymers as soft electrodes for neural bioelectronic interfaces

Luigi Fabiano,[a,b] Tobias Abrahamsson,[a] Ludovico Aloisio,[a] Rémy Cornuéjols,[a] Donghak Byun,[a] Grazia Maria Lucia Messina,[b] Xenofon Strakosas, [a] Daniel T. Simon, [a] Magnus Berggren, [a,c] Chiara Musumeci. [a,*]

[a] Laboratory of Organic Electronics, Department of Science and Technology, Linköping University, 60174 Norrköping, Sweden.

[b] Department of Chemical Sciences, University of Catania and CSGI, Viale A. Doria 6, 95125 Catania, Italy.

[c] Wallenberg Initiative Materials Science for Sustainability, Department of Science and Technology, Linköping University, 60174 Norrköping, Sweden.

*E-mail: chiara.musumeci@liu.se

Monomer synthesis

All reagents and solvents were purchased from commercial suppliers and used as received unless otherwise stated. Tetrahydrofuran (THF) and N,N-dimethylformamide (DMF) were dried overnight over 3 Å and 4 Å molecular sieves, respectively. 2-(2-(2-(trityloxy)ethoxy)ethoxy)ethyl methanesulfonate,[1] 2-(2,5-dibromothiophen-3-yl)ethan-1-ol and 2-(2,3-dihydrothieno[3,4-b][1,4]dioxin-5-yl)-4,4,5,5-tetramethyl-1,3,2-dioxaborolane were synthesized according to previously reported procedures.[2] Flash column chromatography was performed on a Biotage Selekt system using prepacked Sfär HC Duo silica or C18 columns, with samples loaded wet or dry using silica or C18 Samplets®. ^{1}H and ^{13}C NMR spectra were recorded at 25 °C on a Varian Inova-500 Shielded (Oxford AS500) spectrometer operating at 500 and 126 MHz, respectively. Chemical shifts (δ) are reported in ppm and referenced to residual solvent signals of $CDCl_3$ or CD_3OD. NMR data were processed using MestReNova (version 15.1.0-37919).

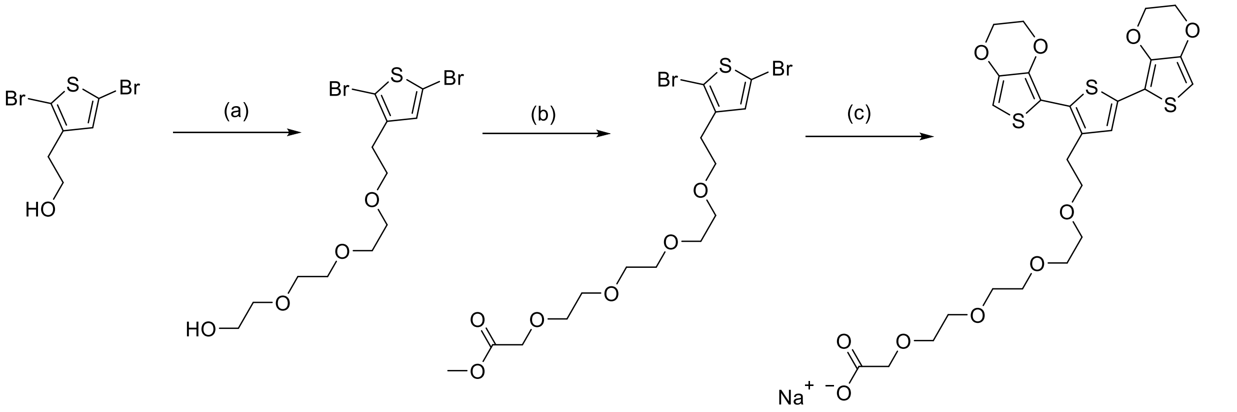

Scheme S1. Synthesis of sodium 14-(2,5-bis(2,3-dihydrothieno[3,4-b][1,4]dioxin-5-yl)thiophen-3-yl)-3,6,9,12-tetraoxatetradecanoate (**ETE-TEG$_3$-C**). (a) NaI (0.1 eq.) in DMF, NaH (1.5 eq.), 30min, 2-(2-(2-(trityloxy)ethoxy)ethoxy)ethyl methanesulfonate (1 eq.), 16h, Dowex® (H-form) in methanol, 60 °C, 2h, 23%. (b) NaI (0.2 eq.), NaH (1.25 eq.) in THF, 30 min, methyl bromoacetate (1.33 eq.), 8h, NaH (1.25 eq.), methyl bromoacetate (1.33 eq.), 16h, r.t., 43%. (c) 2,3-dihydrothieno[3,4-b][1,4]dioxin-5-yl)-4,4,5,5- tetramethyl-1,3,2-dioxaborolane (2.05 eq.), PEPPSI-iPr (5 mol %), 1 M Na_2CO_3 (aq., 2.09 eq.) in THF, 80 °C, 16h, r.t., 1M NaOH (aq. 4.9 eq.), 30 min, 14%.

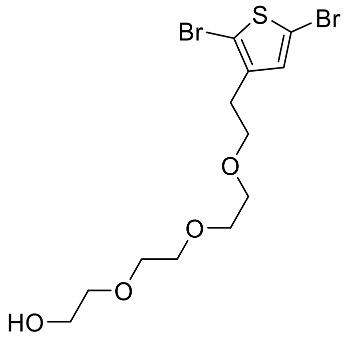

2-(2-(2-(2-(2,5-dibromothiophen-3-yl)ethoxy)ethoxy)ethoxy)ethan-1-ol

2-(2,5-dibromothiophen-3-yl)ethan-1-ol (608 mg, 2.125 mmol) and NaI (32 mg, 0.213 mmol, 0.1 eq.) were dissolved in anhydrous DMF (4 mL) and the solution was purged with N_2-gas. NaH (60%

dispersion in mineral oil) (128mg, 3.200 mmol, 1.5 eq.) was added portion wise at room temperature, and the reaction mixture was stirred for 30 min. A solution of 2-(2-(2-(trityloxy)ethoxy)ethoxy)ethyl methanesulfonate (1000 mg, 2.125 mmol, 1 eq.) in DMF (7 mL) was then added dropwise, and the reaction was allowed to stir at room temperature overnight. The reaction mixture was transferred to a separatory funnel and diluted with DCM (50 mL). The organic phase was washed with water (50 mL), and the aqueous phase was extracted with DCM (50 mL). The combined organic layers were washed with water (2 × 50 mL), dried over anhydrous $MgSO_4$, and filtered. The solvent was evaporated under reduced pressure, and the resulting crude product (≈1.2 g) was dissolved in methanol (30 mL). Dowex® Marathon C resin (H^+-form) (5.25 g) was added, and the mixture was stirred at 60 °C for 2 h. The Dowex resin was removed by filtration, and the solvent was evaporated under reduced pressure. The resulting crude oil was applied directly onto a 20 g Silica HC Duo column and purified by column chromatography using a heptane/ethyl acetate gradient (75:25, 1 CV; 25→100% ethyl acetate, 4 CV), finally eluting the product with 100% ethyl acetate. Product fractions were combined, concentrated under reduced pressure, and dried under high vacuum to afford a viscous oil (200 mg, 23 % yield). ^{1}H NMR (500 MHz, $CDCl_3$) δ 6.83 (s, 1H), 3.70 – 3.51 (m, 14H), 2.94 (b, 1H), 2.74 (t, J = 6.7 Hz, 2H). ^{13}C NMR (126 MHz, $CDCl_3$) δ 139.48, 131.45, 110.30, 108.91, 72.49, 70.57, 70.42, 70.27, 70.17, 69.81, 61.56, 29.83.

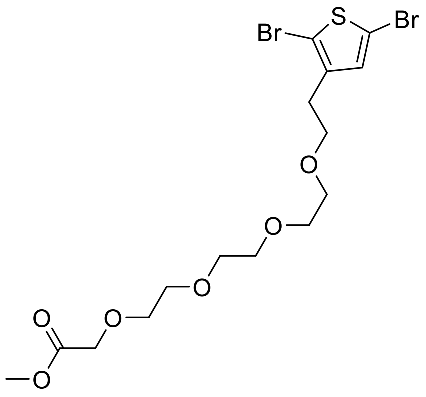

methyl 14-(2,5-dibromothiophen-3-yl)-3,6,9,12-tetraoxatetradecanoate

To an ice cold solution of 2-(2,5-dibromothiophen-3-yl)ethan-1-ol (100 mg, 0.239 mg) in dry THF (2 mL) with NaI (7 mg, 0.048 mmol, 0.2 eq.), was NaH (60% dispersion in mineral oil) (12 mg, 0.300 mmol, 1.25 eq.) added and left to stir for 30 min. Methyl bromoacetate (31 uL, 0.318 mmol, 1.33 eq.) was drop wise added and the reaction was left to stir for 8h at room temperature. Additional NaH (12 mg) and methyl bromoacetate (31 uL) were then added and the reaction was left to stir overnight. The solvent was removed under reduced pressure and the remains were transferred to a separatory funnel with DCM (25 mL) and washed with water (25 mL). The water phase was extracted with DCM (25 mL) and the combined organic phases were dried with $MgSO_4$ and filtered. The crude was concentrated under reduced pressure and applied on a Silica 5g HC Duo column and the product was isolated using an ethyl acetate/acetone gradient (95:5→ 60:40, 7 CV). Product fractions were pooled and the solvents evaporated, yielding a clear yellow-brown viscous oil (50 mg, 43% yield). ^{1}H NMR (500 MHz, $CDCl_3$) δ 6.88 (s, 1H), 4.15 (s, 2H), 3.91 – 3.38 (m, 17H), 2.79 (t, J = 6.7 Hz, 2H).^{13}C NMR (126 MHz, $CDCl_3$) δ 170.98, 139.68, 131.61, 110.48, 109.07, 71.04, 70.76, 70.74, 70.69, 70.53, 70.40, 69.99, 68.74, 51.88, 30.05. ^{13}C NMR (126 MHz, $CDCl_3$) δ 170.98, 139.68, 131.61, 110.48, 109.07, 71.04, 70.76, 70.74, 70.69, 70.53, 70.40, 69.99, 68.74, 51.88, 30.05.

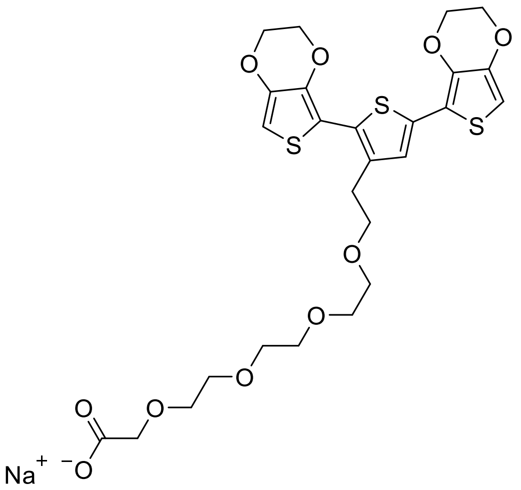

*Sodium 14-(2,5-bis(2,3-dihydrothieno[3,4-b][1,4]dioxin-5-yl)thiophen-3-yl)-3,6,9,12-tetraoxatetradecanoate **(ETE-3TEG-C)***

In a vial containing a solution of methyl 14-(2,5-dibromothiophen-3-yl)-3,6,9,12-tetraoxatetradecanoate (50 mg, 0.102 mmol), 2,3-dihydrothieno[3,4-b][1,4]dioxin-5-yl)-4,4,5,5-tetramethyl-1,3,2-dioxaborolane (80% purity) (70mg, 0.210 mmol, 2.05 eq.) and PEPPSI-iPr (4 mg, 5 mol%) in THF (2 mL), was 1M Na_2CO_3 (aq.) (213 uL, 2.09 eq.) added. The reaction solution was sparged with nitrogen gas, the reaction vial capped and heated to 80 °C overnight. After cooling to room temperature, 1 M NaOH (aq., 0.5 mL) was added and the reaction mixture was stirred for 30 min. The mixture was then transferred to a separatory funnel, diluted with ethyl acetate (25 mL), and extracted with 0.1 M NaOH (aq., 2 × 25 mL). The combined aqueous phases were washed with ethyl acetate (50 mL) and subsequently acidified with 1 M HCl (aq.) until a turbid solution formed. The mixture was then extracted with ethyl acetate (2 × 40 mL) and the combined organic phases were washed with water (40 mL). The solvents were removed under reduced pressure, and the residue was dissolved in THF (2 mL). The crude product was deprotonated by addition of 0.1 M NaOH (aq., 2 mL). The resulting solution was dry loaded onto a C18 silica plug and the product was isolated by column chromatography on a 6 g C18 silica HC Duo column, using a water/acetonitrile gradient (90:10, 2CV; 90:10 → 0:100, 7 CV). The combined fractions were pooled, and the solvents were co-evaporated with methanol under reduced pressure. The residue was then dried under high vacuum overnight to afford the final product (9 mg, 14%). ^{1}H NMR (500 MHz, MeOD) δ 7.13 (s, 1H), 6.45 (s, 1H), 6.30 (s, 1H), 4.38 – 4.31 (m, 2H), 4.31 – 4.26 (m, 2H), 4.26 – 4.19 (m, 4H), 3.86 (s, 2H), 3.72 (t, J = 7.0 Hz, 2H), 3.68 – 3.53 (m, 12H), 2.96 (t, J = 7.0 Hz, 2H). ^{13}C NMR (126 MHz, MeOD) δ 177.22, 143.49, 143.24, 139.76, 139.15, 137.85, 135.40, 128.18, 126.11, 112.80, 110.62, 100.07, 97.82, 72.04, 71.31, 71.14, 70.96, 70.90, 70.88, 70.86, 70.62, 66.37, 66.15, 65.85, 65.75, 30.56.

NMR-Spectroscopy

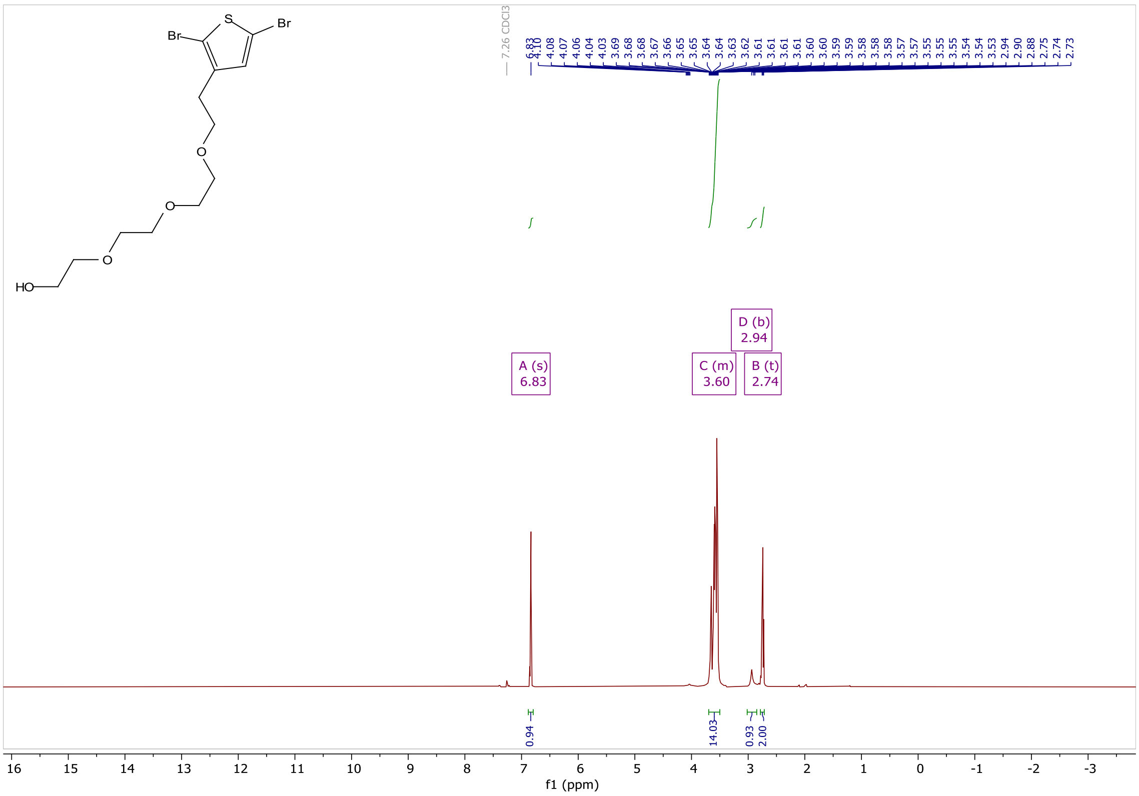

Figure S1. ^{1}H-NMR (chloroform-*d*) spectrum of 2-(2-(2-(2-(2,5-dibromothiophen-3-yl)ethoxy)ethoxy)ethoxy)ethan-1-ol.

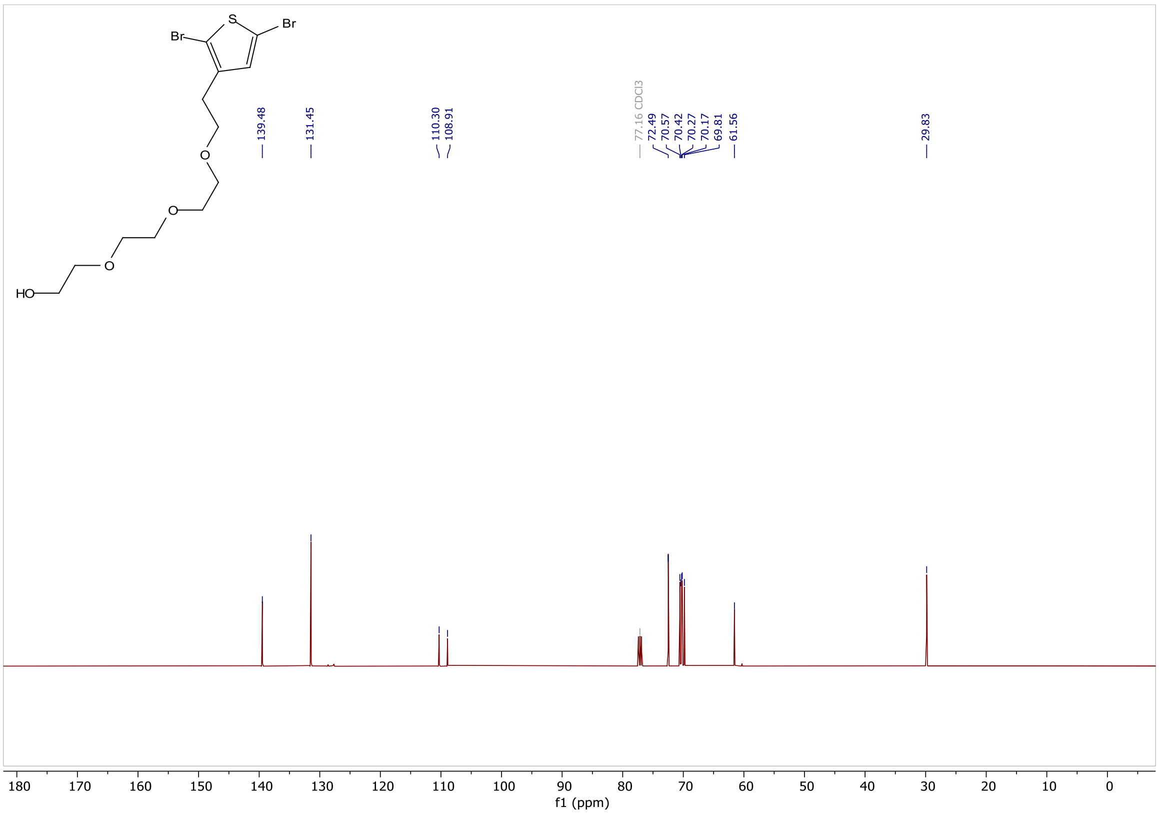

Figure S2. ^{13}C-NMR (chloroform-*d*) spectrum of 2-(2-(2-(2-(2,5-dibromothiophen-3-yl)ethoxy)ethoxy)ethoxy)ethan-1-ol.

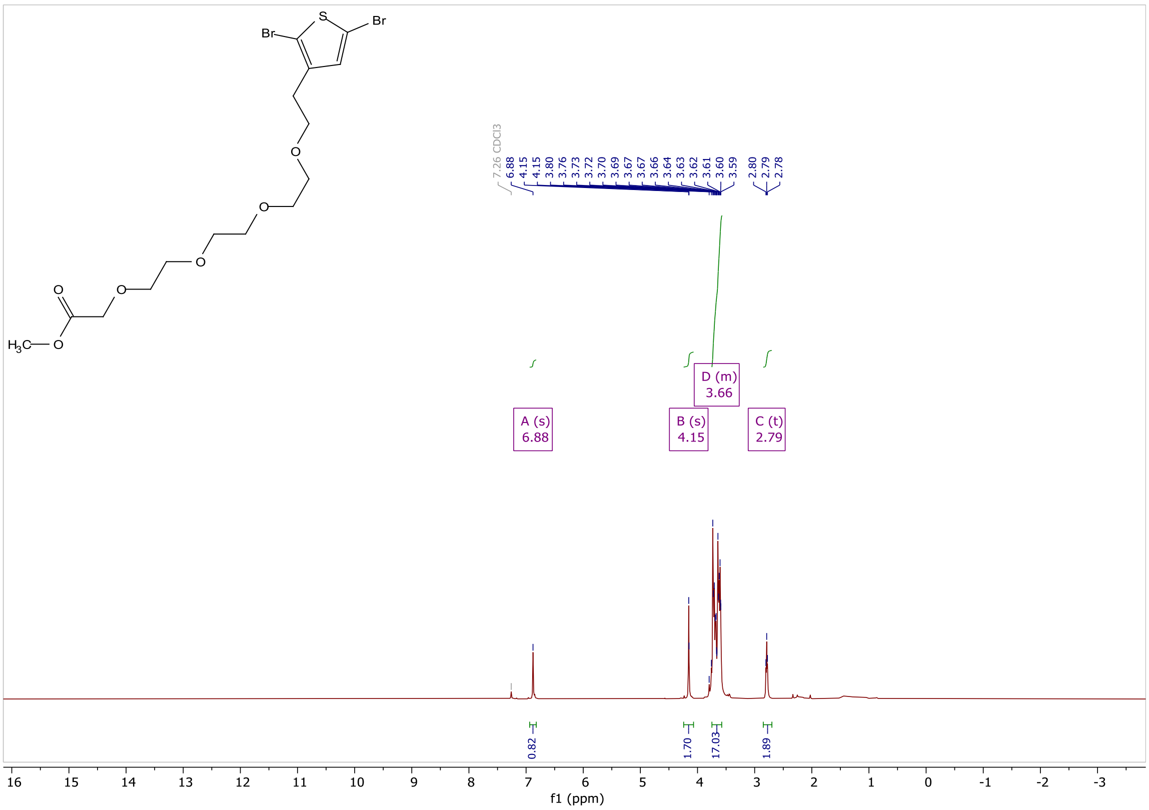

Figure S3. ^{1}H-NMR (chloroform-*d*) spectrum of methyl 14-(2,5-dibromothiophen-3-yl)-3,6,9,12-tetraoxatetradecanoate.

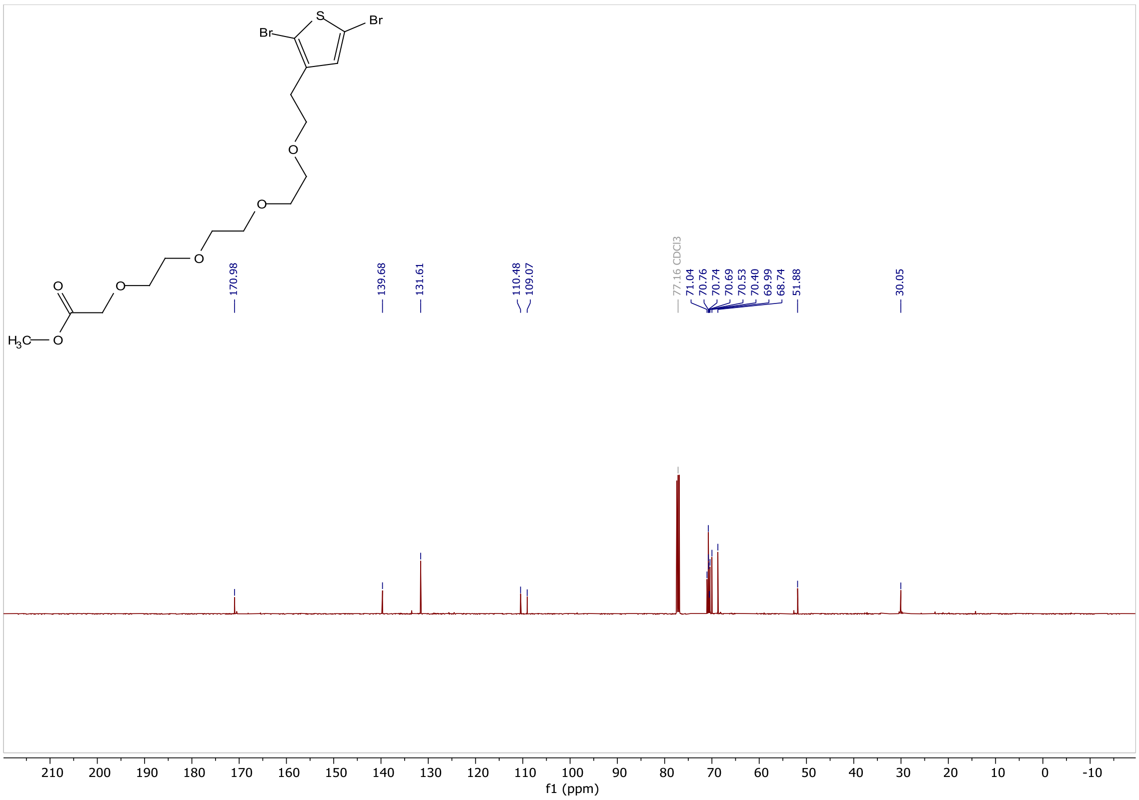

Figure S4. ^{13}C-NMR (chloroform-*d*) spectrum of methyl 14-(2,5-dibromothiophen-3-yl)-3,6,9,12-tetraoxatetradecanoate.

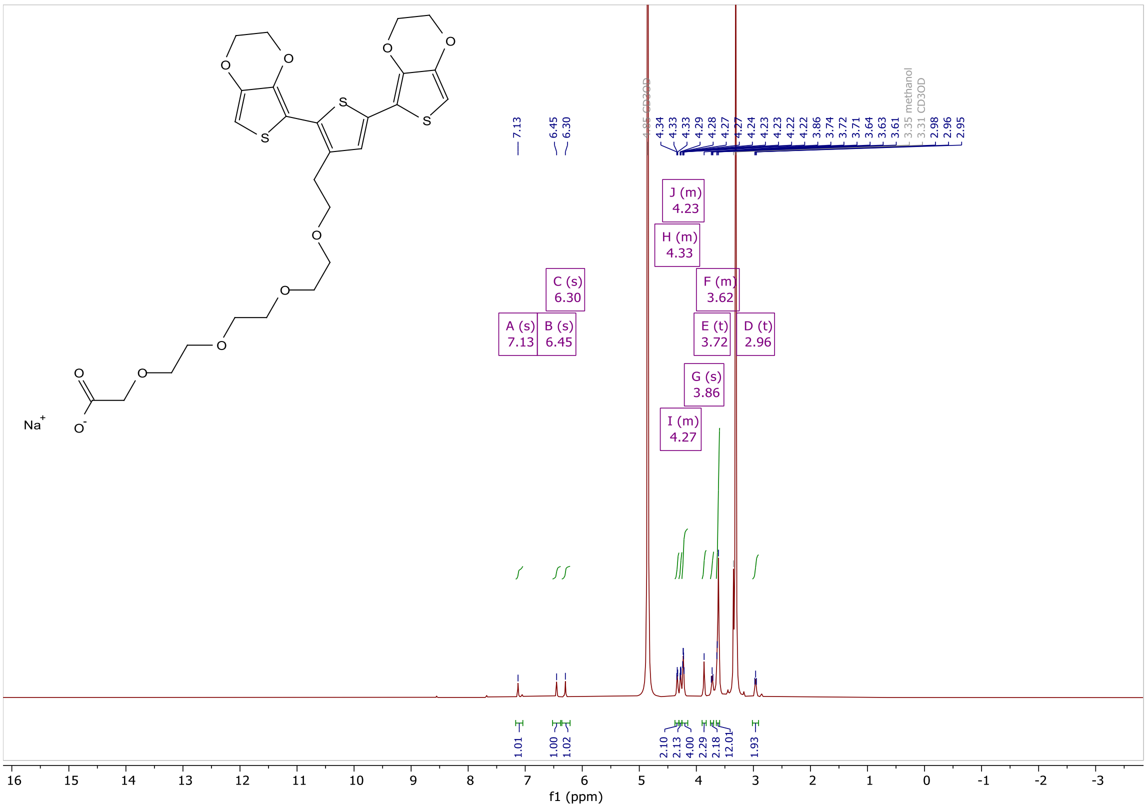

Figure S5. ^{1}H-NMR (methanol-d_4) spectrum of sodium 14-(2,5-bis(2,3-dihydrothieno[3,4-*b*][1,4]dioxin-5-yl)thiophen-3-yl)-3,6,9,12-tetraoxatetradecanoate, ETE-3TEG-C.

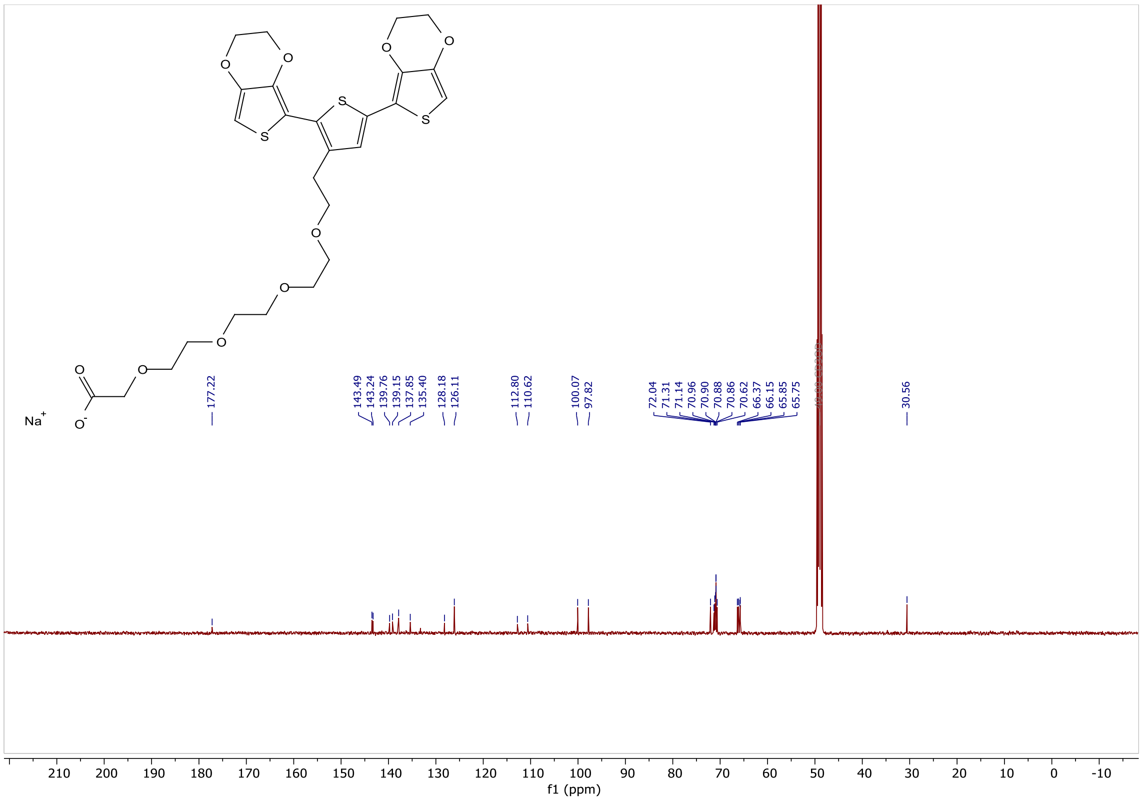

Figure S6. ^{13}C-NMR (methanol-d_4) spectrum of sodium 14-(2,5-bis(2,3-dihydrothieno[3,4-*b*][1,4]dioxin-5-yl)thiophen-3-yl)-3,6,9,12-tetraoxatetradecanoate, ETE-3TEG-C.

Polymer synthesis

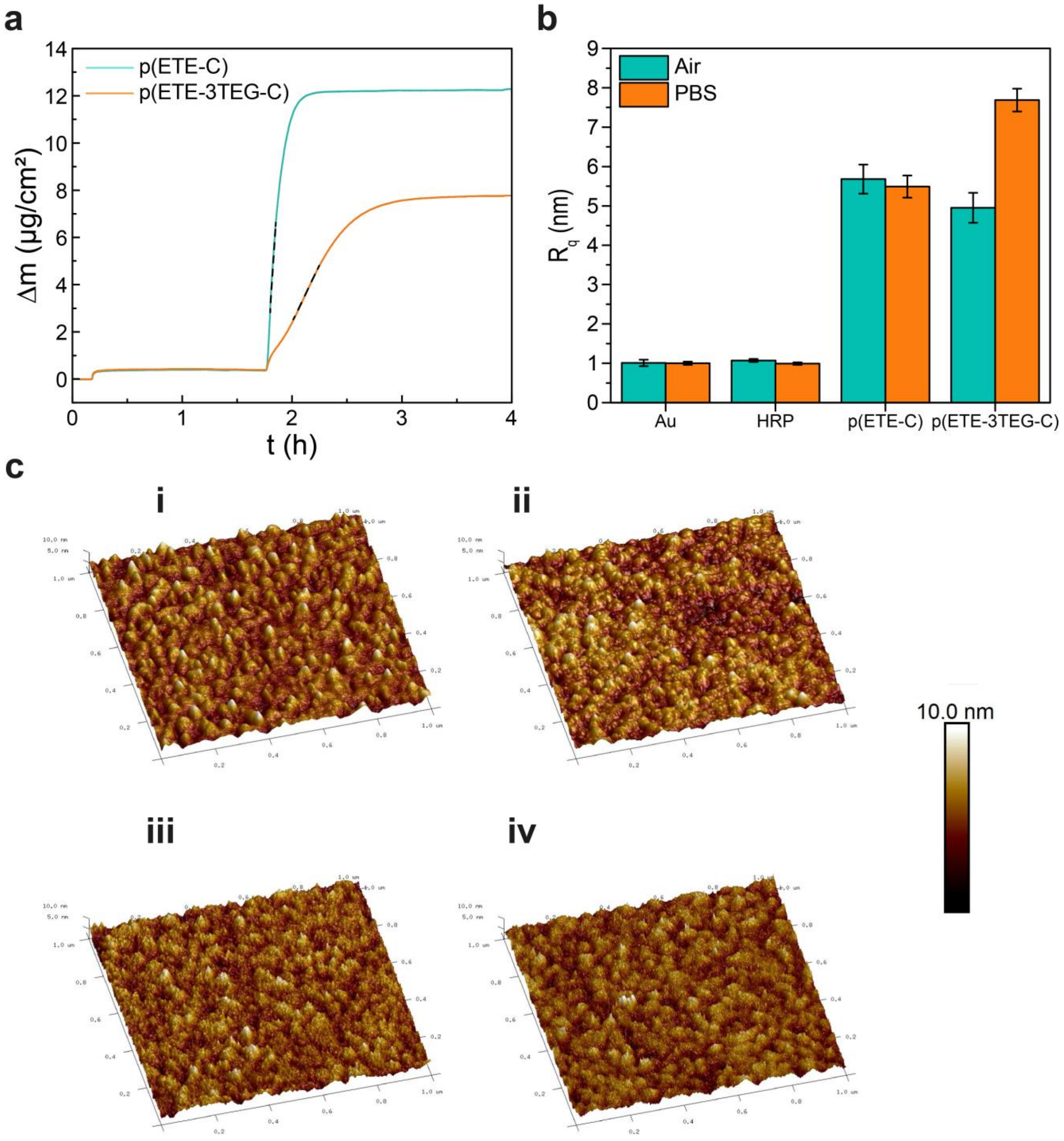

Figure S7. (a) Time evolution of the adsorbed mass (Δm) during the enzymatic polymerization of p(ETE-C) and p(ETE-3TEG-C) on HRP-modified gold surfaces. The linear fitting of the main polymerization region is shown as a black dashed line. (b) Root-mean-square roughness (R_q) of the

Au surface before and after HRP adsorption, measured in air and PBS. Both bare Au and the HRP adlayer display low roughness values and minimal differences between air and PBS. (c) AFM topography images (1 × 1 μm²) of the surfaces at the two initial assembly steps: (i-ii) bare Au in air and PBS, and (iii-iv) HRP-modified Au in air and PBS.

AFM images in Fig. S7c illustrate the morphological evolution of the sensor surface prior to polymer growth. The bare gold electrode displays the characteristic nanograined texture typical of evaporated Au films, with uniform grains and narrow height distribution. Upon HRP adsorption, the surface becomes smoother and more homogeneous, indicating the formation of a thin, conformal protein layer coating the underlying gold grains. The reduction in apparent roughness and the disappearance of sharp grain boundaries confirm efficient enzyme coverage and uniform film formation, providing a stable and reproducible interface for subsequent enzymatic polymerization. No significant differences were observed between measurements in air and PBS for either bare Au ($p = 0.62$) or HRP-coated substrates ($p = 0.08$), confirming that neither surface undergoes hydration-driven rearrangement.

Table S1. Kinetics parameters for p(ETE-C) and p(ETE-3TEG-C) film formation.

Polymer	k (ng cm^{-2} min^{-1})	R^2
p(ETE-C)	795.79±2.95	0.992
p(ETE-3TEG-C)	161.78±0.07	0.999

The kinetic constants reported in Tab. S1 were obtained by fitting the linear portion of the frequency-time trace ($\Delta f(t)$) recorded at the 7th overtone (35 MHz). This overtone was selected because it provides an optimal compromise between sensitivity to surface processes and reduced influence from bulk liquid loading. For both polymers, the slope of the Δf signal in the initial linear growth regime was used to determine the apparent film growth rates.

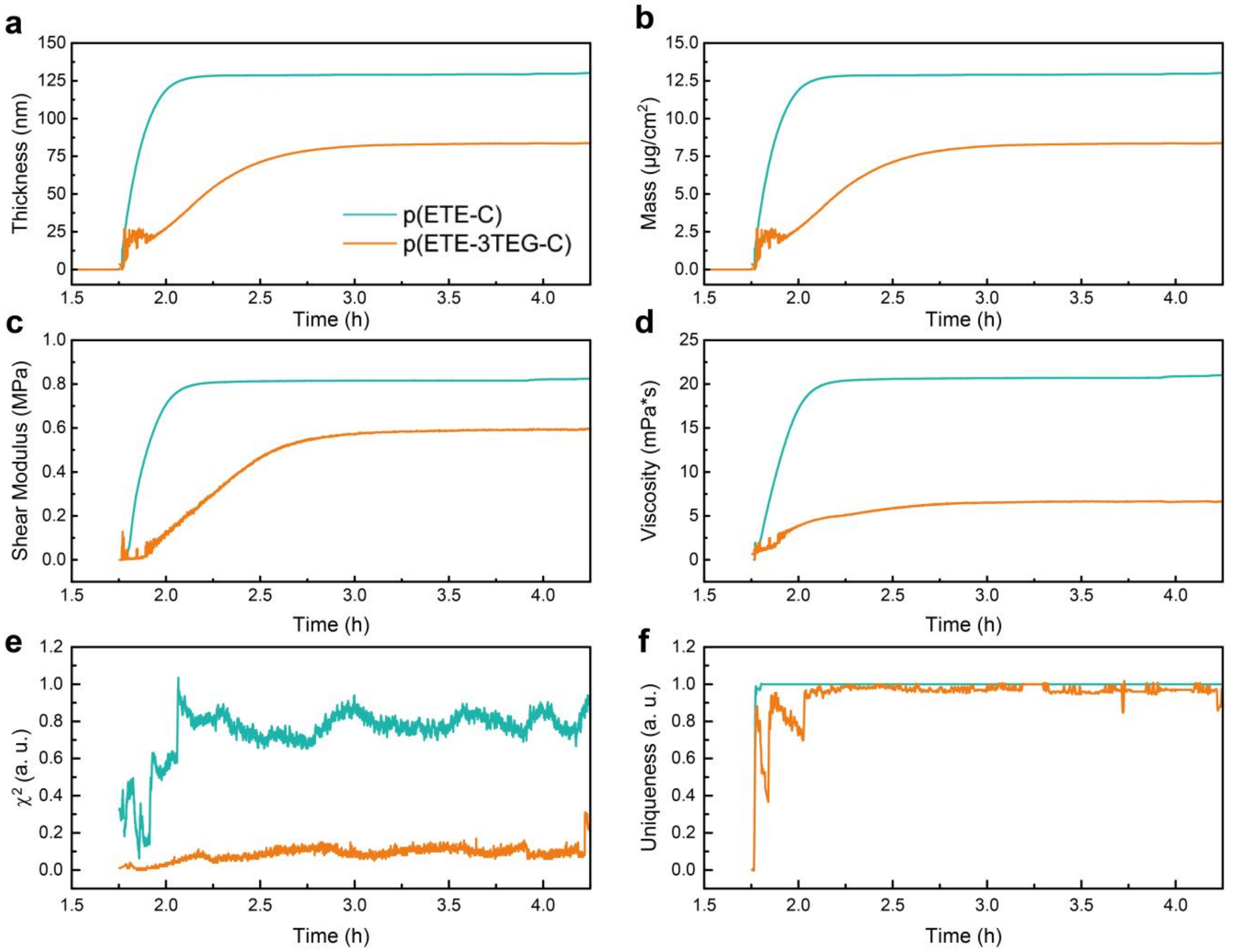

Figure S8. Time-evolution of the viscoelastic properties of p(ETE-C) and p(ETE-3TEG-C) films during enzymatic polymerization, obtained from Voigt VE modelling of QCM-D data. (a) Film thickness h; (b) Areal mass Δm; (c) Shear modulus μ; (d) Shear viscosity η; (e) Reduced chi-square χ^2; (f) Uniqueness index of the fit. p(ETE-C) forms a thicker, denser and mechanically stiffer film, while the presence of the triethylene glycol side chain in p(ETE-3TEG-C) leads to slower growth, lower mass uptake and a softer, more hydrated viscoelastic network. The lower χ^2 values for p(ETE-3TEG-C) indicate a better agreement between the Voigt model and experimental overtones, whereas both materials reach a Uniqueness of about 1 after the onset of growth, confirming the robustness and independence of the fitted viscoelastic parameters.

Table S2. VE model results for p(ETE-C) and p(ETE-3TEG-C) synthesis. Thickness, areal mass, shear modulus, viscosity, and Young Modulus (E) calculated from μ are reported.

	h (nm)	Δm (ng cm^{-2})	μ (MPa)	η (mPa s)	E (MPa)
p(ETE-C)	129.66±0.37	12.97±0.04	0.82±0.01	20.86±0.13	2.13±0.03
p(ETE-3TEG-C)	83.38±0.19	8.34±0.02	0.59±0.01	6.63±0.02	1.53±0.03

The QCM-D analysis reveals clear differences in the viscoelastic behavior of the two polymers (Fig. S8). p(ETE-C) rapidly forms a thick and compact film ($h = 129.66$ nm; $\Delta m = 12.97$ μg cm^{-2}) with

higher shear modulus and viscosity ($\mu = 0.82 \pm 0.01$ MPa; $\eta = 20.86 \pm 0.13$ mPa s), consistent with a dense and rigid network. p(ETE-3TEG-C), instead, grows more slowly and yields a softer and more hydrated layer, as reflected by its lower viscoelastic parameters ($\mu = 0.59 \pm 0.01$ MPa; $\eta = 6.63 \pm 0.02$ mPa s). The goodness-of-fit indicators support the reliability of the modelling: p(ETE-3TEG-C) shows lower χ^2, while both materials reach Uniqueness of about 1, confirming well-defined and independent fitted parameters. Young's moduli derived from μ,[4] ($E = 2.13 \pm 0.03$ MPa for p(ETE-C) and 1.53 ± 0.03 MPa for p(ETE-3TEG-C)) agree with AFM force-spectroscopy measurements, validating the consistency of the QCM-D analysis and nanoscale mechanical probing.

Cell-polymer interfaces

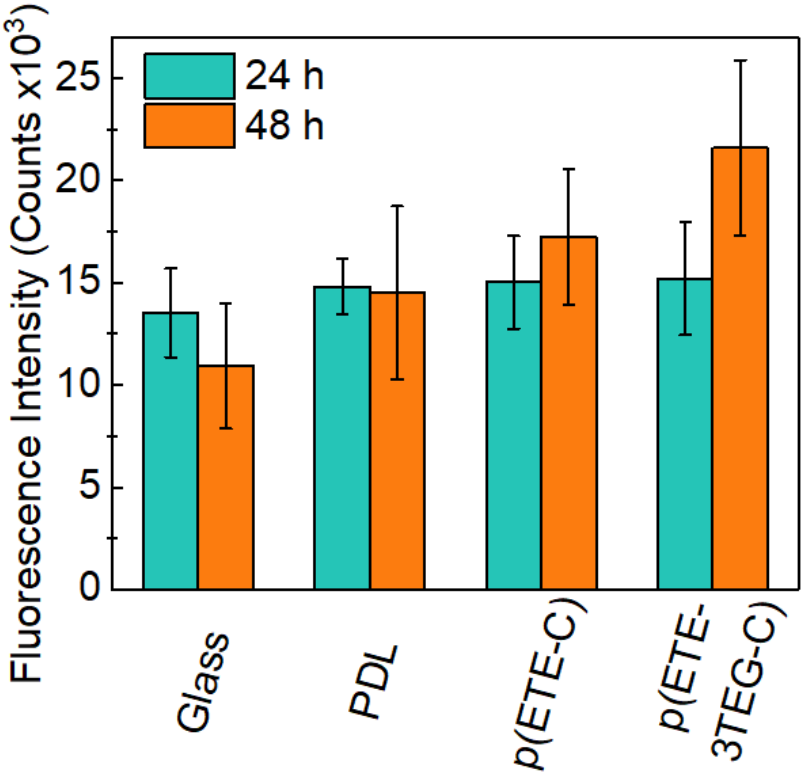

Figure S9. Emission intensity at 600 nm (excitation wavelength: 546 nm) of AlamarBlue after 3 h incubation with F11 cells in complete culture medium, measured at 24 h and 48 h after seeding on Glass, PDL, p(ETE-C), and p(ETE-3TEG-C).

Group	Live (n)	Dead (n)	Total cells (N)	Viability (%)	Mortality (%)
Glass	477	18	495	96.4	3.6
PDL	319	22	341	93.5	6.5
p(ETE-C)	646	61	707	91.4	8.6
p(ETE-3TEG-C)	743	15	758	98.0	2.0

Table S3. Summary of live and dead cell counts for the different substrates (Glass, PDL, p(ETE-C), and p(ETE-3TEG-C)), with total counted cells (N) and the corresponding viability and mortality percentages calculated from the pooled counts.

EC-AFM Nanomechanical Analysis

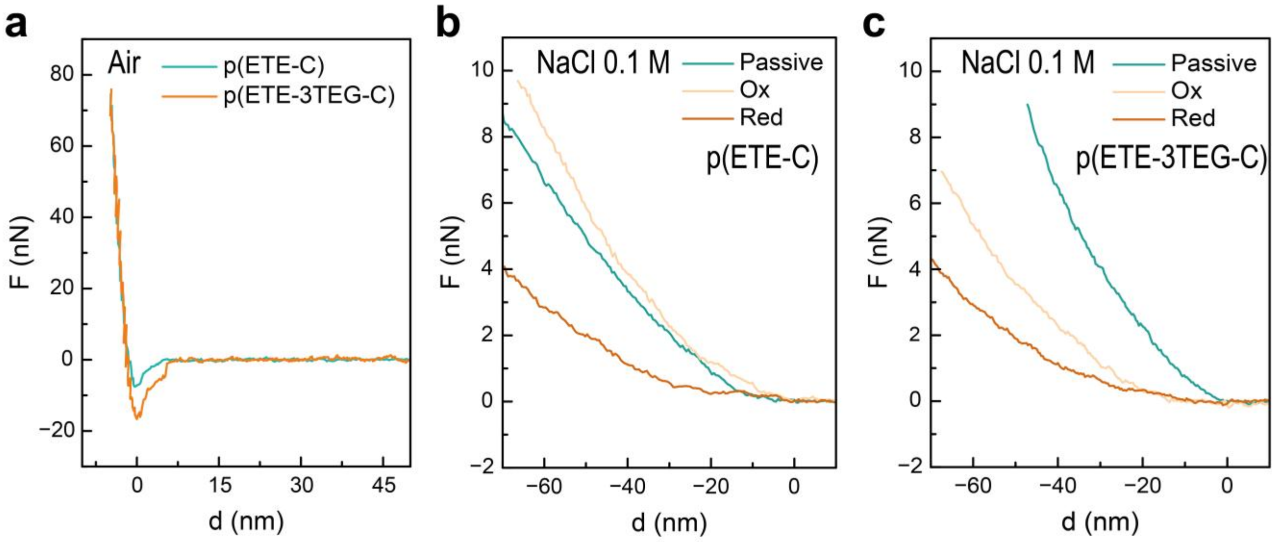

Figure S10. AFM mechanical characterization of p(ETE-C) and p(ETE-3TEG-C) films. (a) Representative force-distance curves acquired in air; (b, c) Force-distance curves recorded in 0.1 M NaCl under passive, oxidized at +0.5 V (Ox) and reduced at -0.5 V (Red) states, highlighting the decrease in stiffness upon reduction.

Table S4. Young Modulus for p(ETE-C) and p(ETE-3TEG-C) across the redox steps. Values are reported as mean value ± SD.

	Young Modulus (MPa)	
Step	p(ETE-C)	p(ETE-3TEG-C)
Air	2026.67±318.95	1635.00±346.48
Passive	3.24±0.69	3.74±1.40
Ox	3.79±1.62	5.18±2.18
Red	1.95±0.68	3.01±0.99
Ox	4.37±2.85	3.77±1.09
Red	2.87±1.06	2.77±0.76
Ox	4.77±3.01	2.81±0.76
Red	3.07±1.37	2.18±0.88

AFM force-spectroscopy reveals a pronounced decrease in Young's modulus when the polymers transition from the dry state to passive swelling in the electrolyte solution: both p(ETE-C) and p(ETE-3TEG-C) drop from GPa values in air to only a few MPa once hydrated. Upon immersion, counter-ions and water enter the film to balance the pre-existing charges, generating a swollen and compliant network.

Most of the mechanical softening occurs already upon hydration, while voltage-driven ion exchange modulates the modulus only modestly.[3] The resulting values closely match those obtained from QCM-D viscoelastic modelling, confirming that hydration and charge compensation dominate the mechanical response of both materials.

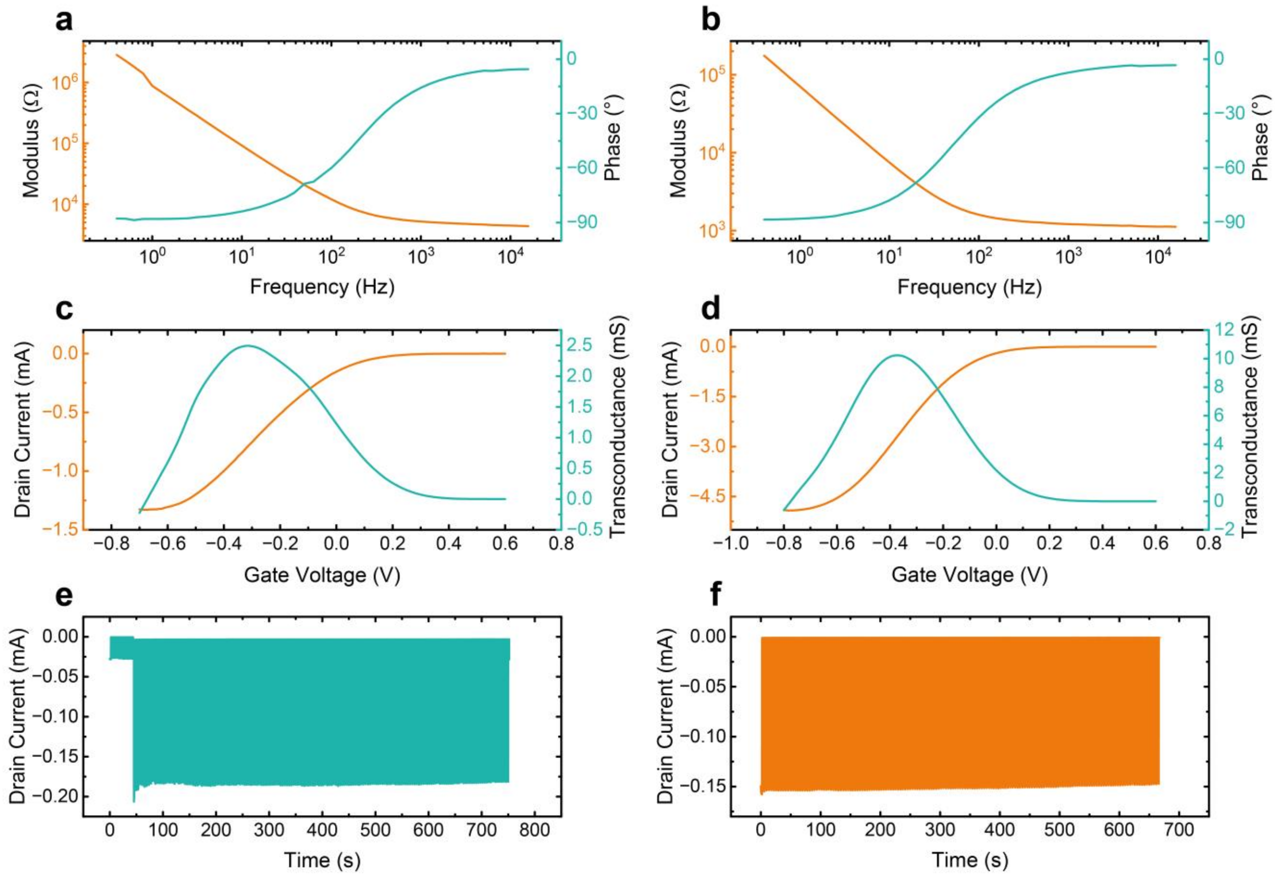

Figure S11. Electrical characterization of OECTs based on p(ETE-C) (left) and p(ETE-3TEG-C) (right), fabricated without a PLL adhesive layer. (a,b) EIS response (modulus and phase vs frequency). (c,d) Transfer curves and transconductance. (e,f) Time-dependent drain current under pulsed gate bias (−0.2/+0.2 V).

References

(1) Janardhanan, J. A. *et al.* Sensitive detection of sweat cortisol using an organic electrochemical transistor featuring nanostructured poly(3,4-ethylenedioxythiophene) derivatives in the channel layer. *Anal. Chem.* **94**, 7584–7593 (2022).

(2) J. Y. Gerasimov, D. Zhao, A. Sultana, T. Abrahamsson, S. Han, D. Bliman, D. Tu, D. T. Simon, R. Olsson, X. Crispin, M. Berggren, S. Fabiano, A Biomimetic Evolvable Organic Electrochemical Transistor. *Adv. Electron. Mater.* 2021, 7, 2001126.

(3) Kumar Paleti, S. H.; Kim, Y.; Kimpel, J.; Craighero, M.; Haraguchi, S.; Müller, C. Impact of Doping on the Mechanical Properties of Conjugated Polymers. *Chem. Soc. Rev.* **2024**, *53* (4), 1702–1729. https://doi.org/10.1039/D3CS00833A.

(4) Barber, J. R. Elasticity; Solid Mechanics and Its Applications; Springer Netherlands: Dordrecht, 2010; Vol. 172. https://doi.org/10.1007/978-90-481-3809-8.
